# Integrated Transcriptomic, Proteomic, and Metabolomic Analyses Revealed Molecular Mechanism for Salt Resistance in Soybean (*Glycine max* L.) Seedlings

**DOI:** 10.3390/ijms252413559

**Published:** 2024-12-18

**Authors:** Siqi Fu, Lu Wang, Chunqian Li, Yinhui Zhao, Nan Zhang, Lei Yan, Chang Ming Li, Yusheng Niu

**Affiliations:** 1 Institute of Biomedical Engineering, College of Life Sciences, Qingdao University, Qingdao 266071, China; fuussq@163.com (S.F.);; 2 Institute of Materials Science and Devices, School of Materials Science and Engineering, Suzhou University of Science and Technology, Suzhou 215009, China

**Keywords:** multiomics, abiotic stress, RNA-seq, DNA methylation, cuticle biosynthesis

## Abstract

Salt stress poses a significant challenge to plant growth and restricts agricultural development. To delve into the intricate mechanisms involved in soybean’s response to salt stress and find targets to improve the salt resistance of soybean, this study integrated transcriptomic, proteomic, and metabolomic analyses to explore the regulatory networks involved in soybean salt tolerance. Transcriptomic analysis revealed significant changes in transcription factors, hormone-related groups, and calcium ion signaling. Notably, the biosynthetic pathways of cutin, suberine, and wax biosynthesis play an important role in this process. Proteomic results indicated salt-induced DNA methylation and the enrichment of phosphopyruvate hydrase post-salt stress, as well as its interaction with enzymes from various metabolic pathways. Metabolomic data unveiled the synthesis of various metabolites, including lipids and flavonoids, in soybean following salt stress. Furthermore, the integrated multiomics results highlighted the activation of multiple metabolic pathways in soybean in response to salt stress, with six pathways standing out prominently: stilbenoid, diarylheptanoid, and gingerol biosynthesis; carotenoid biosynthesis; carbon fixation in photosynthetic organisms; alanine, aspartate, and glutamate metabolism; thiamine metabolism; and pyruvate metabolism. These findings not only offer valuable insights into leveraging multiomics profiling techniques for uncovering salt tolerance mechanisms but also identify candidate genes for soybean improvement.

## 1. Introduction

Soil salinization has emerged as a global concern, as approximately 20% of the world’s total arable land and 33% of the irrigated agricultural land are subject to the adverse impacts of high salinity [1]. Salinity leads to a reduction in total crop yields ranging from 50 to 80% [2,3], thereby resulting in significant economic losses across millions of hectares of land worldwide each year [4]. The intensification of soil salinization, exacerbated by climate change and human activities, has led to an expanding area of affected land [5]. The heat stress resulting from global climate change has led to the scarcity of groundwater and the accumulation of salt levels in the soil. In addition, extensive human irrigation leads to a shallow groundwater table and elevated salinity levels in the topsoil. It is estimated that by 2050, nearly 50% of the world’s arable land will transform into saline–alkali land [6]. Given that crop yields decrease linearly as soil salinity escalates [7], this will significantly reduce crop yields and pose a substantial threat to global food security.

Soybean [*Glycine max* (L.) Merr.] is a vital cash crop. Among all other cereal crops, it has the highest protein content (40–42%) and is one of the largest sources of animal protein feed in the world [8], providing 69% of the world’s daily plant protein consumption [9]. Among edible beans, its oil content is second only to peanuts (18–22%) [10], accounting for about 30% of the world’s edible oil crops [11]. China, facing a prolonged inadequacy in soybean production, has seen soybean imports surpassing 90 million tons in recent years, with an alarming 85% dependence on foreign sources. In 2017, China imported about 95.5 million tons of soybeans, while its total production was only 14.3 million tons [12]. With population growth, food production needs to increase by at least 70% by 2050 to meet demand [13]. Therefore, studying the salt tolerance of soybean is essential for enhancing the utilization of saline–alkali soil and consequently, increasing yield. This initiative not only contributes to local agricultural resilience but also aligns with global food security efforts.

Salt stress typically inflicts damage on plants through mechanisms such as osmotic stress, ionic poisoning, and secondary damage [14]. Osmotic stress and ionic toxicity are considered to be separate in time and space. In the first stage of osmosis, the rapid increase in salt concentration around the root system leads to a significant decrease in the growth rate [15,16]. Osmotic stress arises from the low water potential surrounding plant roots, resulting in cell dehydration and exerting an impact on cell division and expansion, which ultimately influences plant growth and development [15]. The osmotic signal represents a physical signal that cannot interact with the components within the cell directly. At present, it is believed that osmotic stress may cause biophysical changes in plant cells in many aspects, including destruction of cell wall integrity, changes in membrane tension, cell damage, and macromolecular aggregation. However, it remains unclear which of these changes can activate the osmotic signal [17,18,19]. In the second stage, the growth is further reduced due to the accumulation of salt in the old leaves to toxic levels [15,16].The primary sources of salt stress are neutral salts, including NaCl and Na_2_SO_4_, which directly penetrate cells via channels and carrier proteins [14], for example, non-selective cation channels (NSCC), high affinity potassium transporters (HKT), voltage-independent channels (VIC), Aquaporin, etc., leading to ionic toxicity [20]. After NaCl treatment of soybean for 24~72 h, the intracellular Na^+^ concentration increased to a toxic level [21]. In addition, salt stress can disrupt the redox metabolic balance in plant cells, and the oxidative production of plant cells mainly includes reactive oxygen species (ROS), reactive nitrogen (RNS), and reactive sulfur (RSS) [22], resulting in oxidative stress. With the continuation of oxidative stress, the redox state of plant cells is more disordered, and irreversible oxidative damage occurs to functional macromolecules (lipids, etc.), and the metabolism is further damaged, and even leads to the death of plant individuals.

To date, the salt tolerance mechanisms of soybean can be categorized into four groups: maintaining ionic homeostasis, regulating osmotic stress, restoring oxidative balance, and other metabolic and structural adaptations [11]. Various signal-transduction pathways are activated in response to ion stress induced by salt stress, with the well-studied plant salt excretion channel being the salt overly sensitive (SOS) pathway. In Arabidopsis (*Arabidopsis thaliana*), the SOS pathway serves as a typical signal-transduction system for maintaining ion homeostasis [14]. Additionally, plant hormone-mediated, Ca^2+^-dependent, and phosphatidylinositol signaling pathways are also triggered [23,24]. These signaling pathways activate other regulators, such as transcription factors (TFs), to amplify gene regulatory signals, ultimately initiating protective mechanisms by inducing or inhibiting functional genes [25]. To address initial osmotic stress, plants regulate water potential and maintain cell swelling by adjusting stomatal conductance and increasing intracellular osmotic concentration [15]. Furthermore, plants synthesize organic substances with large molecular weights, like sugars and alcohols, to enhance the osmotic potential of cells, thereby improving their ability to absorb water in a hypertonic environment. Previous studies have shown that the overexpression of *GmNAC06* induces the accumulation of proline and betaine, scavenges excess ROS, and protects the integrity of cell membranes. At the same time, it maintains ion homeostasis and osmotic equilibrium by regulating the transport of Na^+^ and K^+^ [26]. The expression of soybean homologous gene *J* of *AtELF3* regulated the expression of *GmWRKY12*, *GmWRKY27*, *GmWRKY54*, *GmNAC11*, and *GmSIN1* to a certain extent, and improved the salt tolerance of soybean [27]. Despite a series of pathways and genes associated with salt tolerance in soybean having been characterized, the regulatory network of soybean response to salt stress remains unclear.

In recent years, the use of omics technology to investigate the impact of abiotic stress on plants and their response mechanisms has become an effective technical approach [28]. At the molecular level, plants respond to stress through alterations in gene expression, protein abundance, and metabolite accumulation [29]. Transcriptome analysis has emerged as a highly effective method for exploring genome-wide gene expression reprogramming in response to various stresses [30]. Currently, several salt-responsive genes and molecular regulatory pathways have been identified in soybean seedlings [31,32]. Proteomics serves as a potent molecular tool for characterizing the complete proteome at the organelle, cell, organ, or tissue level, and for comparing how the proteome is influenced by diverse physiological conditions [33]. This approach has been applied to plant research in species such as potato (*Solanum tuberosum*) [34], tomato (*S. lycopersicum*) [35], tobacco (*Nicotiana tabacum*) [36], and Arabidopsis [37]. Moreover, proteomics has been utilized to study salt-responsive proteins in rice (*Oryza sativa*), revealing that an oxygen-evolving enhancer protein expressed in the leaf sheath and leaves of rice demonstrates a coordinated response to salt stress [38]. Metabolomics is an emerging approach in genomics research that focuses on detecting metabolic phenotypes and their direct correlation with genotypes under specific conditions. Utilizing metabolics, studies have been conducted to uncover the metabolic changes in various plant species, including soybean [39], maize (*Zea mays*) [40], wheat (*Triticum aestivum*) [41], tomato [42], Arabidopsis [43], and tobacco [44] under different stresses.

Transcriptome studies exclusively focus on the mRNA levels. Nevertheless, multiple post-transcriptional regulatory mechanisms exist [45]. Consequently, the data obtained from transcriptome studies fails to comprehensively represent all the responses of plants to salt stress, and subsequently, it may not align with the findings obtained from proteomic analysis [46]. The accumulation of plant metabolites is intricately linked to plant growth and regulated by environmental factors [47]. Proteins and their intricate interactions govern the biosynthesis of compounds crucial for growth or defense. Consequently, the metabolome introduces an additional layer of complexity to plant adaptive responses, potentially influencing both the proteome and/or transcriptome [48]. Therefore, relying solely on single omic analysis provides only partial insights into the mechanisms employed by certain plants to combat abiotic stresses. A comprehensive understanding of how plants navigate abiotic stresses necessitates a multifaceted approach. Currently, the mechanism through which plants respond to abiotic stress has been unveiled by integrating transcriptome and metabolome data. This approach has been successfully applied to various plant species, including *Sophora alopecuroides* [28], soybean [49], and tobacco [50]. Moreover, integrated multiomics have revealed the drought-tolerance mechanisms in rice [51], the abiotic-resistance mechanism in tomato [52], and the drought-tolerance mechanism in soybean [53]. Unfortunately, there is a dearth of research employing the combined analysis of three omics to investigate salt tolerance in soybean. In this study, we employed an integrated approach that incorporating transcriptomic, metabolomic, and proteomic investigations to delve into the molecular mechanisms underlying salt tolerance in soybean. This, in turn, provides additional knowledge for breeding and cultivation of salt-tolerant soybean varieties.

## 2. Results

### 2.1. Morphological and Physiological Responses of Soybean to Salinity Treatment

**Figure 1:**
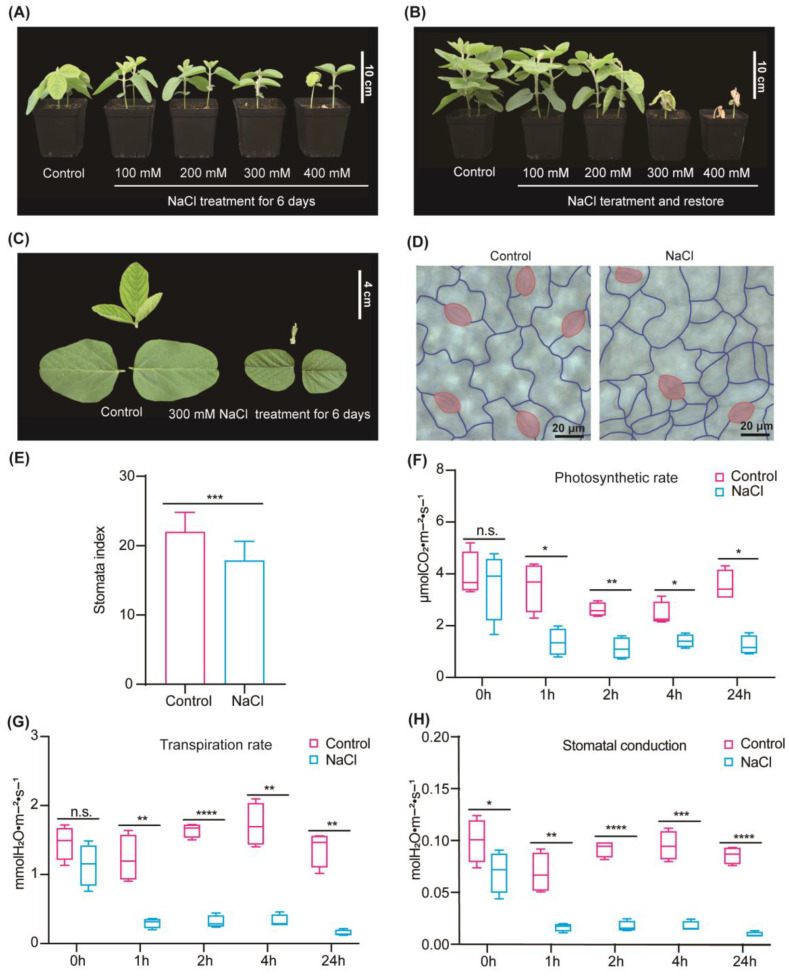
Phenotype and physiological response of soybean seedlings under salt stress. (**A**) Phenotype of soybean seedlings treated with different concentrations of NaCl for 6 days. (**B**) Soybean seedlings were treated with different concentrations of NaCl for 11 days and then recovered their phenotype for 6 days. (**C**) Phenotype of soybean seedlings treated with 300 mM NaCl for 6 days. (**D**) Stomatal phenotype of soybean seedlings treated with 300 mM NaCl for 6 days. Stomata are shown in red, and the pavement cells are outlined in blue. (**E**) Stomata index of soybean seedlings treated with 300 mM NaCl for 6 days, *t* test, n.s.: *p* > 0.05; *: *p* ≤ 0.05; **: *p* ≤ 0.01; ***: *p* ≤ 0.005; ****: *p* ≤ 0.001. Each bar represents the means ± SD, *n* = 15. (**F**–**H**) Photosynthetic rate (**F**), Transpiration rate (**G**), Stomatal conductance (**H**), *t* test, n.s.: *p* > 0.05; *: *p* ≤ 0.05; **: *p* ≤ 0.01; ***: *p* ≤ 0.005; ****: *p* ≤ 0.001. Each bar represents means ± SD, *n* = 4.

Under various concentrations of salt stress, soybean seedlings exhibited distinct morphological and physiological changes in their leaves. Following 1, 2, and 4 days of salt stress, seedlings subjected to different salt concentrations displayed varying degrees of inhibition (see Figure S1). Notably, soybean seedlings treated with 100 mM and 200 mM NaCl exhibited less growth inhibition. In contrast, the seedlings treated with 300 mM and 400 mM NaCl presented obvious growth inhibition. Meanwhile, those exposed to 400 mM NaCl displayed yellowing of leaves on the fourth day. The results on the sixth day of salt stress showed that only the leaves of 400 mM NaCl treatment were wilted and curled (Figure 1A). After 11 days of salt stress, 6-day recovery period was granted to the soybean seedlings (Figure 1B). The seedlings subjected to 100 mM and 200 mM NaCl demonstrated recovery, whereas those under 300 mM and 400 mM NaCl failed to regain normalcy. Interestingly, the soybean leaves exposed to 300 mM NaCl for 1, 2, 4, and 6 days exhibited significant growth inhibition, however, without reaching a lethal degree (Figure 1C). Therefore, the concentration of 300 mM NaCl was selected in the following studies.

Upon subjecting soybean seedlings to 300 mM NaCl for 6 days, a notable reduction in stomatal density was observed (Figure 1D,E). Comparative analysis between treated and untreated seedlings revealed a sharp decline in the photosynthetic rate, stomatal conductance, and transpiration rate from 1 h to 24 h, respectively (Figure 1F–H). Elevated salinity leads to a decrease in the environmental water potential, resulting in osmotic stress on plants and triggering a cascade of osmotic responses in soybean plants to counter this “physiological drought”. This assertion is supported by the observed decrease in stomatal conductance subsequent to the 300 mM NaCl treatment.

### 2.2. Alterations in Transcriptome Profiles in Response to Salt Stress in Soybean

To explore how cultivated soybean responds to salt stress, we exposed soybean plants to 300 mM NaCl. Eighteen pair-end libraries were established, encompassing five different time points and a control, each replicated thrice. RNA-seq results are summarized in Table S1. A total of 4694 differentially expressed genes (DEGs) were identified in response to salt stress. The complete list of all DEGs is available in Tables S2–S6. After 2 h of salt stress, 382 genes were upregulated, and 141 genes were down-regulated. Following 4 h of salt stress, there were 945 upregulated genes and 704 downregulated genes. After 12 h post-salt stress, 1651 genes were upregulated, and 1148 genes were downregulated. At 24 h of salt stress, there were 112 upregulated genes and 410 downregulated genes. Finally, at 48 h of salt stress, 611 genes were upregulated and 583 genes were downregulated (Figure S2A). Among these DEGs, 22 genes were consistently differentially expressed at all five time points (Figure S2B).

#### 2.2.1. Functional Enrichment of DEGs

**Figure 2:**
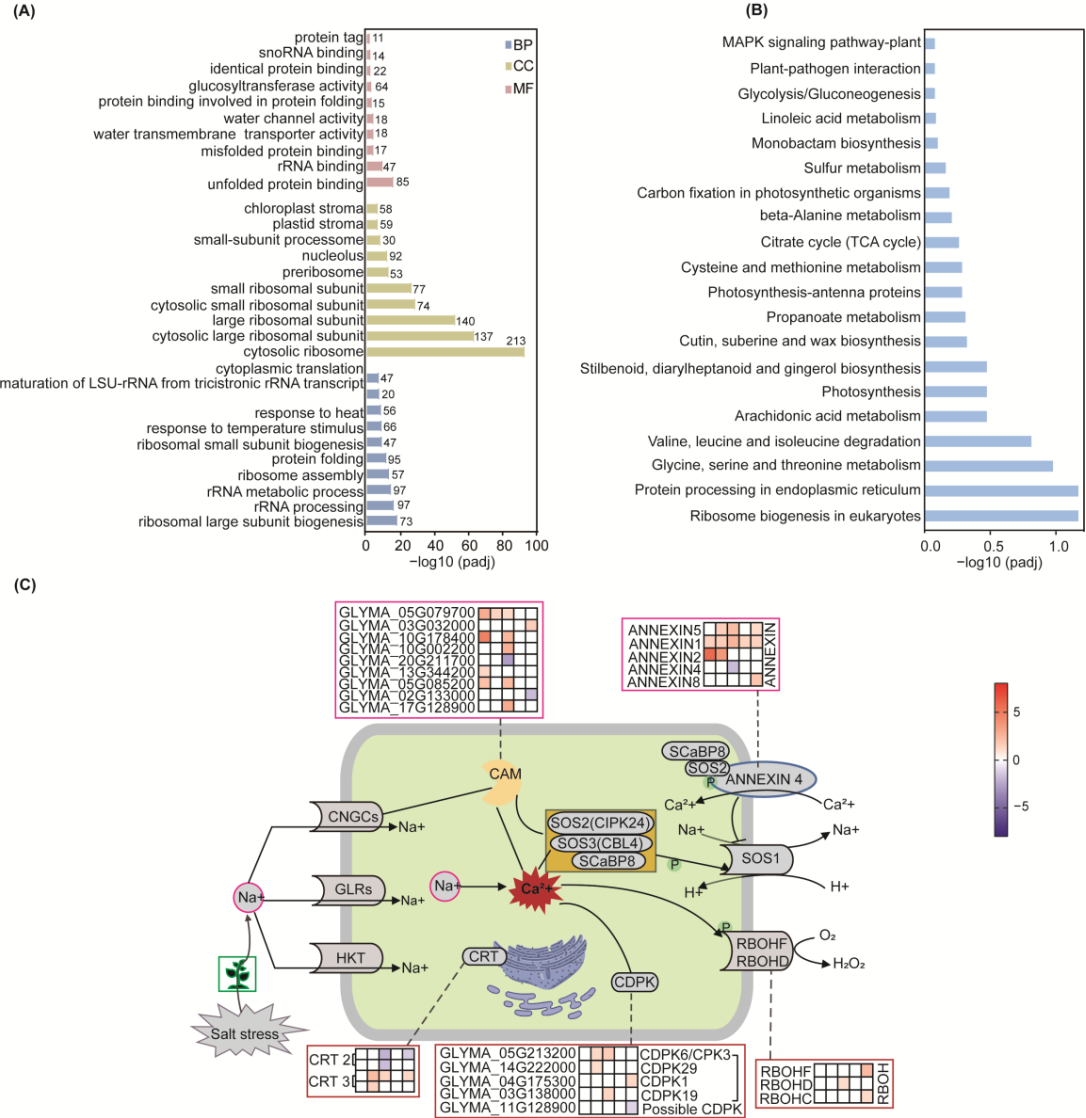
A total of 300 mM NaCl induces functional enrichment and validation of the transcriptome. (**A**) GO functional enrichment of DEGs after salt stress. In the figure, the ordinate is GO Term, the abscissa is the significance level of GO Term enrichment, which is represented by − log_10_ (padj), and different colors represent different functional classifications (BP, MF, CC). (**B**) KEGG pathway enrichment of DEGs after salt stress. The ordinate is the KEGG pathway, and the abscissa is the significance level of pathway enrichment. (**C**) Links and changes between calcium ion signal-transduction pathways and plant hormones and transcription factors after salt stress. Ca^2+^ signaling plays a crucial role in various aspects of plant salt stress response. Salt-sensitive receptors include SOS1 (salt overly sensitive), Na^+^/H^+^ antiporters, histidine kinases, AHK1/ATHK1 (Arabidopsis Histidine Kinase), and NSCC (nonselective cation channels). Arabidopsis NSCCs are mainly divided into two categories: cyclic nucleotide-gated channels (CNGCs) and glutamate activation channels (GLRs). Salt-induced Ca^2+^ signaling activates the SOS pathway and enhances plant salt tolerance, and SOS3/SCaBP8, the main part of the pathway, is the calcium ion-binding protein of EF-hand, which decodes and transmits calcium signaling. Upon receiving the signal, SOS3/sCaBP8 interacts with the terminal regulatory domain of the SOS2 protein, thereby activating SOS2 to form the SOS3/sCaBP8-SOS2 complex. The complex then triggers the activation of the Na^+^/H^+^ anti-transporter SOS1 on the plasma membrane, facilitating Na^+^ efflux through phosphorylation modification. Furthermore, the SOS2-phosphorylated SCaBP8 formation complex interacts with the Ca^2+^-dependent membrane-bound protein Annexin4 to regulate Ca^2+^ signaling under salt stress with the SCaBP8-AtANN4-SOS2 complex and improve the Na^+^/H^+^ transport activity of the plasma membrane. Moreover, CDPKs play a role in multiple plant signaling pathways downstream of elevated Ca^2+^ concentrations, thereby regulating various physiological responses. Calreticulin (CRT) is a calcium-binding protein of the endoplasmic reticulum (ER) that has a variety of functions in addition to its role as a chaperone. Plant CRT has antioxidant effect in transgenic plants, which alleviates the oxidative stress of plants and improves the stress resistance of plants. In plants, “respiratory burst oxidase homologous” (RBOH) proteins localize to the plasma membrane and have been reported to regulate various biological processes, including pathogen responses and abiotic stress tolerance, through the ability of ROS to regulate production as a second messenger. Under salt stress, Ca^2+^ in the cytoplasm transiently activates CAM protein, which opens the signaling network through the calcineurin pathway, thereby attenuating salt-induced damage caused by high Na^+^ levels. Gradient colors indicate log_2_-fold changes (FC) in gene expression in leaves at different time points (2, 4, 12, 24, and 48 h) compared to controls (0 h).

To comprehensively elucidate the functional and biological processes of DEGs in soybean under salt stress, we performed Gene Ontology (GO) function enrichment analysis of DEGs. We examined enriched GO terms in three categories: Biological Process (BP), Cellular Component (CC), and Molecular Function (MF) at each time point (Table S7). We integrated all transcriptome data, selecting and mapping the top 10 distinct GO terms in the three categories (Figure 2A; complete data in Table S8). The GO results of BP showed that the response of plants to oxidative stress was enhanced, including the response to reactive oxygen species and cellular redox homeostasis. The effect of soybean on osmotic stress was also correspondingly enhanced, and some oligosaccharide synthesis-related genes were also significantly upregulated, such as raffinose. In addition, a large number of genes associated with phytohormone stress were also significantly upregulated, particularly in response to abscisic acid (ABA) and ethylene (ET). In contrast, a large number of genes related to developmental functions were significantly downregulated, including reproductive development, flower development, and cell wall production. Many genes associated with photosynthesis were significantly downregulated, including photosynthesis and light harvesting. The results also showed that related genes such as glycolysis and pyruvate metabolism were downregulated. The MF results showed that the genes related to GO functions such as calcium ion binding, signal receptor binding, and ethylene receptor binding were upregulated, while most of the genes related to transmembrane transport, methyl transfer, and glycosyl transfer were significantly downregulated. The cellular component category is more likely to identify genes related to the cell wall and many organelle genes that are significantly downregulated, including thylakoids, chloroplasts, ribosomes, etc. In addition, photosystem-related functional genes were also significantly downregulated. Furthermore, we integrated all transcriptome data and identified the top 20 KEGG pathways that exhibited significant differences (Figure 2B). The complete data set is available in Table S9. Our KEGG pathway analysis revealed that the DEGs identified in soybean seedlings following salt stress were primarily enriched in amino acid metabolism, glycolysis, photosynthesis, carbon fixation in photosynthetic organisms, MAPK signaling pathway, and plant hormone signaling.

Our results showed that metabolic pathways such as photosynthesis and glycolysis were disrupted after salt stress, which may be one of the reasons for the limitation of soybean growth and development. The influence of cell wall and plasma membrane disruption led to osmotic stress after salt treatment, and redox-related gene enrichment indicated that soybean was subjected to oxidative stress. Soybeans respond to salt stress by activating some signaling pathways or synthesizing metabolites, such as raffinose, diphenyl compounds, diaryl heptanes, and gingerol organisms. This is consistent with the coordination of different organelles, such as endoplasmic reticulum, chloroplasts, mitochondria, peroxisomes, and nucleus, working together to maintain cell viability and homeostasis through material communication and information transfer, as observed in our GO enrichment results.

#### 2.2.2. Response of Plant Hormones After Salt Stress

Plant hormones are crucial small molecules that regulate various aspects of plant growth and development. Among them, ABA, ethylen, salicylic acid (SA), and jasmonic acid (JA) are recognized as stress-responsive hormones. To gain further insights, we conducted a comprehensive analysis and heat mapping of DEGs associated with plant hormones. A total of 106 plant hormone-related DGEs were screened, including gibberellin (GA), ethylene, ABA, Auxin, and JA (Figure S2C). Specifically, we identified a total of 13 genes related to GA. Among these, three genes are orthologs of the rice GA receptor gene (*OsGID1*), which are GLYMA_03G148300, GLYMA_10G022900, and GLYMA_20G230600. GLYMA_06G043400 encodes the F-box protein involved in GA signaling, and these four genes are upregulated after salt stress. Four genes encoding gibberellin oxidase or proteins with oxidase activity, which are GLYMA_13G259400, GLYMA_13G218200, GLYMA_16G200800, and GLYMA_20G141200, have been identified. Additionally, a gene-encoding member of the CYP701A cytochrome P450 family, GLYMA_15G002200, has also been identified that was downregulated after salt stress.

Regarding ethylene, we found 11 genes associated with ET-related processes. Among these, three genes encoded a nuclear transcription factor that initiates the downstream transcriptional cascade of ethylene reactions, namely GLYMA_13G076700, GLYMA_14G041500, and GLYMA_20G051500. In addition, the ETR2 types that have been shown to be involved in the sensing of ethylene in Arabidopsis were screened, which were GLYMA_20G202200 and GLYMA_10G188500. The identified ethylene-related DEGs were upregulated after salt stress.

In the case of AUXIN, we detected 43 genes associated with this plant hormone. Among these, 15 genes were SAUR/SMALL AUXIN UPREGULATED RNA (SAUR) gene families, including nine SAUR14 genes, and the relative contents were all upregulated after salt stress. There were three SAUR32 classes, of which two genes were upregulated. The relative expression of two SAUR78 genes and one SAUR50 gene were downregulated after salt stress. In addition, six DEGs were members of the auxin response factor family, with four ARF8s, one ARF9, and one ARF16.

Furthermore, we identified nine genes related to JA whose relative contents were upregulated after salt stress. Among these, GLYMA_14G078600 is a member of the cytochrome p450 CYP74 gene family, and the relative content of cytochrome p450 was upregulated by eightfold after 2 h of salt stress.

Among the 30 ABA-related genes, four DEGs encode PYR/PYL/RCAR family proteins, which function as ABA sensors, and these four DEGs were downregulated after salt stress. Additionally, two DEGs encoding Protein phosphatase 2C (PP2C) were upregulated after salt stress, and five DEGs encoding ABI FIVE-binding protein 3 (AFP3) were identified to be upregulated after salt stress. GLYMA_03G213300 encodes a membrane-bound protein called AtTSPO, which was significantly induced after salt stress, and this gene was upregulated 1775-fold after 12 h of salt stress. GLYMA_20G158500 and GLYMA_10G236000 encode a protein that is induced to be expressed in response to water scarcity (e.g., cold, high salt, and dryness), and these two genes were upregulated 91-fold and 865-fold, respectively, at 12 h of salt stress. Our results suggest that the accumulation of plant hormones or the activation of related signaling pathways after salt stress can mitigate the damage caused by salt stress. However, the specific mode of action of certain genes requires further investigation.

#### 2.2.3. Response of Transcription Factors After Salt Stress

The ability of plants to tolerate salt is inseparable from the expression of stress-related functional genes regulated by transcription factors, and some transcription factors can alleviate the damage caused by salt stress to plants by regulating the expression of downstream target genes. Our transcriptome results showed significant changes in a large number of transcription factors after salt stress, and we conducted a comprehensive analysis and heat mapping of DEGs associated with transcription factors (Figure S3A). A total of 130 DEGs associated with transcription factors were screened out, exhibiting an increase of more than twofold (Log_2_(FC) > 1). The results showed that 21 of them belonged to the WRKY family, with WRKY33 exhibiting more significant changes; GLYMA_03G042700 was upregulated by 176-fold, and GLYMA_11G163300 by 120-fold after 12 h of salt stress.

A total of 22 DEGs were of the bHLH family, and three DEGs were involved in the plant hormone-mediated signaling pathway, namely GLYMA_01G019700, GLYMA_04G199900, and GLYMA_06G165700. The former was involved in gibberellin-mediated flowering control, and its relative content decreased after salt stress; the latter two were upregulated after salt stress and played a role in the cytokinin signaling pathway. GLYMA_05G110700, GLYMA_05G036800, and GLYMA_09G149900 were significantly upregulated in this family, and 48-fold, 120-fold, and 12-fold were upregulated after salt stress, respectively, but the specific role of these three genes in salt stress remains to be studied.

In addition, 17 DEGs were of the bZIP family, and five of them encoded proteins bound to ABA reaction elements, all of which were upregulated after salt stress. A total of 34 DEGs belonged to the MYB family, and three of them directly activated lignin biosynthesis genes and phenylalanine biosynthesis genes during the formation of secondary walls, which were MYB20 (GLYMA_02G244600), MYB42 (GLYMA_17G133800), and MYB43 (GLYMA_18G040700), respectively. The relative contents of these three DEGs were increased by more than three times, 33 times and 18 times after 12 h of salt stress, respectively. GLYMA_18G201800 was upregulated by 87-fold at 12 h under salt stress, but the specific function of this gene remains to be studied.

There were 36 DEGs belonging to the AP2 family, of which 16 were ethylene reactive factor (ERF) subfamilies, 15 were DREB subfamilies, and four other classes of transcription factors. Fourteen transcription factors of the ethylene response factor (ERF) subfamily were upregulated and two downregulated after salt stress. There was one ERF B-4 subfamily (GLYMA_05G186700) whose relative content was upregulated by 247 times after 2 h of salt stress, five ERF B-1 subfamilies, four B-3 ERF subfamilies, one ERF B-2 subfamily, two ERF B-6 subfamilies, and three ERF B-5 subfamilies. These transcription factors are involved in ethylene signaling cascades, cytokinin localization, JA reactions, etc. Of the 15 DREB subfamily transcription factors, 13 were upregulated and two downregulated after salt stress, including three DREB subfamily A-6, six DREB subfamily A-4, five DREB subfamily A-5, and one DREB subfamily A-2. The results showed that transcription factors accumulated in large quantities after salt stress, and some transcription factors could synergistically regulate the salt tolerance mechanism of plants with plant hormones. Additionally, some transcription factors could improve the salt tolerance of plants by stimulating the formation of secondary walls. Our results screened a large number of unstudied transcription factors, providing many possibilities for future studies of salt tolerance in soybeans.

#### 2.2.4. Response of Calcium Ion Signaling After Salt Stress

Our GO functional enrichment results showed that the calcium ion-binding gene was upregulated after salt stress, and the concentration of Ca^2+^ as an intracellular second messenger increased after salt stress, triggering downstream signaling pathways. Therefore, we performed a more in-depth analysis of the calcium ion signal after salt stress (Figure S3B). A total of 62 DEGs were identified with calcium-binding function, of which 45 were upregulated and 17 were downregulated. There were nine genes encoding calmodulin (CaM), of which seven increased in content and two DEGs were downregulated after salt stress. Additionally, there were four DEGs encoding calreticulin (CRT) and four DEGs encoding calnexin (CANX). Among the five DEGs encoding calcium-binding annexin, one was downregulated while the rest were upregulated; notably, GLYMA_08G136200 encoding annexin 2 was upregulated by more than 53-fold at 2 h under salt stress.

A total of 12 DEGs encoding calcium-binding (CaBPs) EF hand family proteins were recorded, with 2 DEGs downregulated after salt stress and the rest upregulated. Among them, GLYMA_06G258000 increased by 34 times after 12 h of salt stress. The results also showed that four DEGs encoding calcium-dependent protein kinase (CDPK) were upregulated after salt stress, while one was putatively downregulated; all of these were related to the plant hormone ABA metabolic pathway. In addition, 3 DEGs encoding calcium sensors and 3 DEGs encoding respiratory burst oxidase homologous (RBOH) were also upregulated after salt stress. Furthermore, the relative content of DEG (GLYMA_13G035900) encoding wall-associated kinase 5 was upregulated by more than 37-fold after salt stress. Our results innovatively indicate that RBOH may play a regulatory role in the aftermath of salt stress. In conclusion, our findings suggest that calcium ion signaling regulates the damage caused by salt stress in many ways. It not only triggers the “salt oversensitivity” (SOS) pathway but also coordinates certain hormones and transcription factors to regulate plant salt tolerance, achieving plant developmental plasticity (Figure 2C).

### 2.3. Proteomic Responses to Salt Stress in Soybean

To investigate changes in the proteome of soybean seedling leaves after 24 h of 300 mM NaCl treatment, TMT quantitative proteomics was employed. Data from three biological replicates were analyzed, and proteins were detected by searching the soybean protein database. A total of 9290 proteins were identified in six samples, of which 9257 were quantified. Among them, 49 proteins showed significant changes in expression, with 23 proteins being significantly upregulated and 26 proteins being significantly downregulated (Figure S4A; Table S10).

#### 2.3.1. Functional Enrichment of DEPs in Soybean After Salt Stress

**Figure 3:**
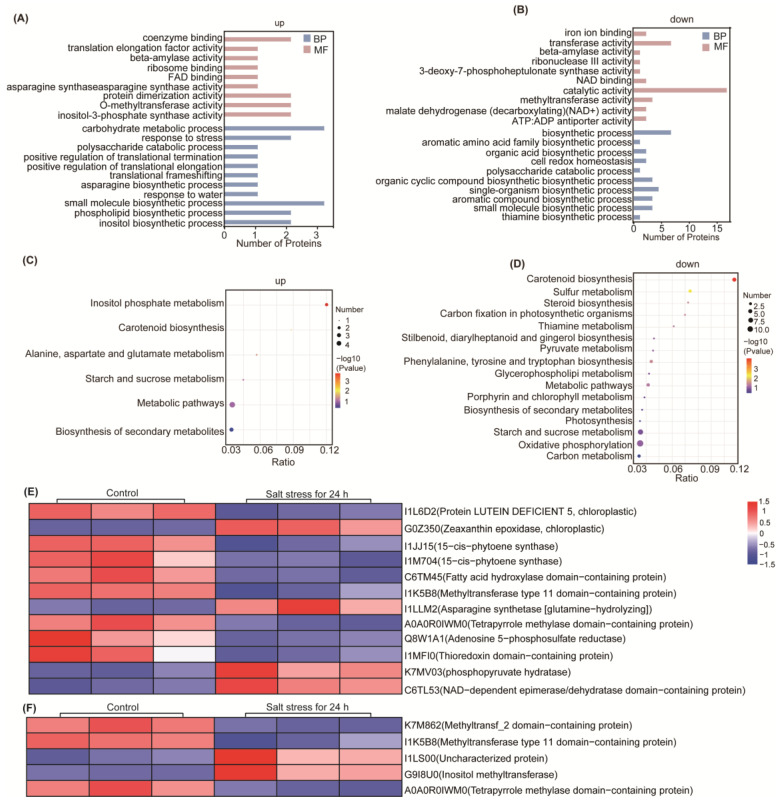
300 mM NaCl induces functional enrichment and validation of the Proteomics. (**A**,**B**) GO functional enrichment of upregulated and downregulated DEPs. In the figure, the ordinate is GO Term, the abscissa is the significance level of GO Term enrichment, which is represented by −log_10_ (padj), and different colors represent different functional classifications (BP, MF, CC). (**C**,**D**) KEGG metabolic pathway enrichment of upregulated and downregulated DEPs. The horizontal coordinate in the graph is x/y (number of differential proteins in the corresponding metabolic pathway/number of total proteins identified in the pathway), and the larger the value, the higher the enrichment of differential proteins in the pathway. The color of the dots represents the *p*-value of the hypergeometric test, with smaller values indicating greater reliability and statistical significance of the test. The size of the dots represents the number of differential proteins/metabolites in the corresponding pathway, and the larger the value, the more differential proteins there are in the pathway. (**E**,**F**) Changes in methyltransferases after 24 h of salt stress (**E**). Changes in protein-protein interactions important node proteins after 24 h of salt stress (**F**). The clustering level of the proteins is shown with the samples grouped vertically, and the colors indicate the normalized converted values of the relative quantification values of the differential metabolites.

Based on the above data, a systematic bioinformatics analysis was performed on all identified DEPs, including protein functional annotation, functional enrichment, and pathway enrichment. GO functional significance enrichment analysis revealed significantly enriched GO functional entries in DEPs compared to all identified protein backgrounds, providing insights into the biological functions that the differential proteins are associated with (Table S11). The main enrichment of DEPs was observed in upregulated processes such as lipid biosynthesis, response to stress, and the activity of some synthases (Figure 3A). Conversely, the enrichment of DEPs was dowregulated in processes related to thiamine and organic acid biosynthesis, aromatic biosynthesis, transferase activity, and catalytic activity (Figure 3B).

KEGG Pathway significant enrichment was used to identify the most important biochemical metabolism pathways and signal-transduction pathways involved in DEPs. The pathways involved in the upregulation of DEPs enrichment include inositol phosphate metabolism, alanine, aspartic acid, and glutamate metabolism (Figure 3C). Conversely, the pathways associated with downregulated DEPs encompassed pyruvate metabolism, glycerol phospholipid metabolism, photosynthesis, and oxidative phosphorylation pathways (Figure 3D). Carotenoid biosynthesis, starch and sucrose metabolism, secondary metabolite biosynthesis, and metabolic pathways were enriched in both upregulated and downregulated DEPs (Table S12).

Understanding the subcellular localization of proteins is crucial for organismal studies. We annotated the subcellular localization of DEPs, with 13 DEPs having specific annotations (Figure S4B). Cytoplasmic and mitochondrial proteins comprised 30.77% each, while microsomal proteins represented the smallest proportion at 7.69%. Nuclear and endoplasmic reticulum proteins each accounted for 15.38% of the annotated DEPs.

The variability in protein domains likely underlies the observed protein differences, highlighting the importance of studying protein domains for understanding their biological function and evolution. A total of 56 domains were identified across all differential expressed proteins, with 35 significantly distinct domains identified based on a *p*-value threshold of less than 0.05. The top 10 domains with the largest differences were shown in Figure S4C, and the full data are shown in Table S13. Notably, the top 10 enriched domains among DEPs encompassed O-methyltransferase family 2, plant methyltransferase dimerization, and adenosine phosphate phosphosulfate reductase (Figure S4C). This enrichment suggests that soybean may undergo specific DNA methylation in response to salt stress, potentially regulating gene expression in response to abiotic stress, thereby modulating soybean’s salt stress response.

Additionally, the enrichment of phosphate synthase and the reduction of adenosine phosphate phosphosulfate reductase following salt stress may signify a response to oxidative stress induced by salt stress. Significant alterations in glycoside hydrolase family 14 and malic enzyme N-terminal also suggest inhibition of several metabolic pathways in soybean post-salt stress. Moreover, the involvement of squalene/phytoene synthase in carotenoid biosynthesis underscores the potential role of carotenoid biosynthesis pathways in soybean’s response to salt stress.

#### 2.3.2. Analysis of Protein-Protein Interactions Among DEPs

To investigate the relationships among the identified DEPs, we utilized the StringDB protein-protein interaction database (http://string-db.org/, accessed on 13 March 2023) and imported the interaction data into Cytoscape software (https://cytoscape.org/) for visualization (Figure S4D; Table S14). Further analysis of proteins at critical nodes (Figure 3E). Our analysis revealed that the interacting proteins were associated with eight metabolic pathways. Notably, four DEPs were found to be involved in the carotenoid biosynthesis pathway, with I1L6D2, G0Z350, I1JJ15, and I1M704 identified as key contributors, where I1JJ15 and I1M704 were the rate-limiting enzymes in this process. Furthermore, protein C6TM45 was implicated in steroid biosynthesis and exhibited significant interactions within the carotenoid synthesis pathways, as well as with the protein I1K5B8 in the glycerophospholipid metabolism pathway. Protein I1LLM2 was associated with the metabolism of alanine, aspartic acid, and glutamic acid, interacting with five proteins and significantly linked to L-asparaginase. Protein A0A0R0IWM0, involved in porphyrin and chlorophyll metabolism, displayed significant interaction with the proteins Q8W1A1 and I1MFI0 within the sulfur metabolism pathway. The upregulated protein K7MV03, a phosphopyruvate hydrase, interacted with enzymes involved in photosynthesis, oxidative phosphorylation, carbon fixation, pyruvate metabolism, and carbon metabolism. Lastly, the upregulated protein C6TL53, containing a NAD-dependent epimerase/dehydrase domain and annotated as a flavonol reductase/cinnamoyl-CoA reductase with a role in defense and resistance, interacted with proteins in carotenoid synthesis pathway, steroid biosynthesis pathway, and amino acid metabolism pathway, as well as proteins containing the enoylreductase (ER) domain. These observed protein-protein interactions strongly suggest that salt stress substantially influences the metabolic pathways in soybean, potentially leading to the synthesis of carotenoids, steroids, and other biologically significant substances as part of the plant’s stress response mechanism.

#### 2.3.3. Responses of DNA Methylation After Salt Stress

Methylation levels in plants are a dynamic process where different genes are activated downstream, participating in various regulatory pathways. Our domain analysis revealed significant changes in methyltransferases following salt stress, prompting further investigation into methyltransfer-related proteins (Figure 3F). We identified five methylation-related proteins: G9I8U0, I1LS00, K7M862, I1K5B8, and A0A0R0IWM0. K7M862 is a Methyltransf_2 domain protein, while I1K5B8 is a methyltransferase type 11 domain-containing protein; both exhibit methyltransferase activity and show a downregulation in relative content after salt stress. In contrast, G9I8U0, an inositol methyltransferase, experiences a 3.4-fold increase in content post salt stress. A0A0R0IWM0, which contains a tetrapyrrole methylase domain, also has methyltransferase activity but is downregulated after salt stress. Notably, I1LS00 is an uncharacterized protein with a methyltransferase domain, and its content is upregulated by 3.4-fold after salt stress.

### 2.4. Metabolomic Responses to Salt Stress in Soybean

**Figure 4:**
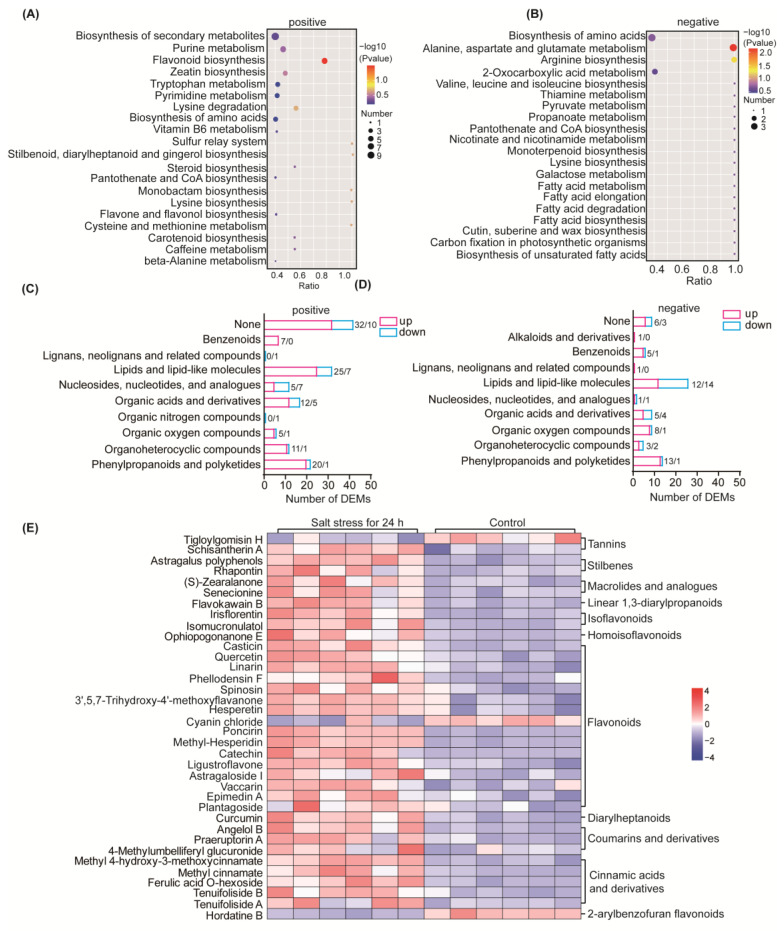
Metabolomic changes in soybean seedlings under salt stress. (**A**,**B**) KEGG metabolic pathway enrichment of positive-ion DEMs and negative-ion DEMs. Same as Figure 3C,D. (**C**) Classification of positive DEMs. (**D**) Classification of negative DEMs. (**E**) Accumulation of Phenylpropanoids and polyketides after 24 h of salt stress. Clustering of metabolites is shown horizontally, samples are grouped vertically, and colors indicate values after normalization transformation of relative quantitative values of differential metabolites.

Untargeted metabolomics technology was applied to study the metabolomic changes in soybean seedling leaves following treatment with 300 mM NaCl for 24 h, as compared to the control, with six replicates for each condition. Among the 152 positive-ion DEMs identified, 117 metabolites were upregulated, while 35 metabolites were downregulated (Figure S5A; Table S15). Among the 82 negative-ion DEMs, 55 metabolites were upregulated, and 27 metabolites were downregulated (Figure S5B; Table S16). The selected DEMs were then subjected to KEGG enrichment analysis, and the KEGG pathways mapped to positive-ion DEMs included flavonoid biosynthesis, steroid biosynthesis, amino acid biosynthesis and metabolism, pantothenic acid and coenzyme A biosynthesis, purine and pyrimidine metabolism, and carotenoid biosynthesis (Figure 4A; Table S17). The KEGG pathways mapped to negative-ion DEMs included fatty acid metabolism and biosynthesis, unsaturated fatty acid biosynthesis, ascorbic acid and aldehyde acid metabolism, citrate cycle (TCA cycle), secondary metabolite biosynthesis, carbon metabolism, cutin, suberine and wax biosynthesis, and some amino acid synthesis and metabolism (Figure 4B; Table S18).

To understand the functional properties and classification of the different metabolites, we annotated the identified metabolites using the Human Metabolome Database (https://hmdb.ca/metabolites, accessed on 25 March 2023), Consequently, with reference to ClassI classification of the identified DEMs, there was a notable presence of phenylpropanoids, polyketides, lipids, and lipid-like molecules in both positive and negative DEMs (Figure 4C,D). Following salt stress, a majority of phenylpropanoids and polyketones demonstrated upregulation, with predominantly increased levels observed in positive DEMs of lipids and lipid-like molecules, while both upregulation and downregulation were noticed in negative ions. Additionally, several non-classified DEMs were identified among the positive DEMs. Further analysis was carried out for the main upregulated metabolite groups “phenylpropanoids and polyketides” (Figure 4E). A total of 22 positive ion metabolites of phenylpropanoids and polyketones were identified, of which 20 metabolites were upregulated, and 14 negative ion metabolites were upregulated, of which 13 were upregulated. The results were reclassified, and two of them were Stilbenes, namely Rhapontin and Astragalus polyphenols, whose relative concentrations were upregulated by more than twofold after salt stress. There were 16 flavonoid metabolites accumulated, of which the content of catechins increased 13-fold after salt stress and Ligustroflavone increased more than twofold. Flavonoids can increase the antioxidant capacity of plants, while other metabolites identified have not been analyzed in detail to explain the relationship with salt tolerance in plants. In addition, D-(-)-Fructose and (±)-Abscisic acid were accumulated in the negative ion DEMs. D-(-)-Fructose is increased by sixfold, which may help to alter the osmotic potential of the cellular environment in response to osmotic stress due to salt stress. Our metabolomic results show that many potential chemicals accumulate after salt stress, and they may be potential exogenous agents to study to improve salt tolerance in soybeans.

### 2.5. Integrative Analysis of Dual Omics Data

We conducted association analyses of paired omics data sets. Transcriptomics and proteomics are valuable tools for investigating the physiological and chemical state of a system, and the combined analysis of these two omics enhances the detection rate of protein identification and validates gene mutation information at the protein level. Metabolomics provides transcriptomics with notably significant data support. Combined analysis of the proteome and metabolome can reduce background noise, streamline the identification of research targets, and elucidate underlying mechanisms. Integrating dual-omics data facilitates a thorough comprehension of biological systems.

#### 2.5.1. Integrative Analysis of Metabolomic and Transcriptomic Data

Correlation analysis was performed based on the Pearson correlation coefficient between DEGs and DEMs (Tables S19 and S20) so as to measure the degree of association between the differential genes and the differential metabolites. A correlation coefficient less than 0 indicates a negative correlation, while a coefficient greater than 0 indicates a positive correlation. Using a correlation coefficient absolute value greater than or equal to 0.95 and a *p*-value ≤ 0.01 from the joint analysis, we identified a total of 14 negative DEMs and 26 positive DEMs that were positively correlated with DEGs. Additionally, 24 positive DEMs and 11 negative DEMs were negatively correlated with DEGs. Among the positively correlated DEMs, there were three positive DEMs {5′-S-methyl-5′-thioadenosine, N-(5-acetamidopentyl) acetamide, and 5-chloro-2,8-dimethyl-4-[(3-nitro-2-pyridyl)oxy)] and two negative DEMs (thiamine phosphate and D-Saccharic acid) correlated with no fewer than 200 DEGs each, all downregulated after salt stress. Conversely, three positive DEMs (N’2-(2-furylcarbonyl)-3-chloro-4-metylthiophene-2-carbohydrazide, isomucronulatol, and praeruptorin A) and one negative DEM (vaccarin) were negatively correlated with no fewer than 200 DEGs each, which were upregulated after salt stress.

**Figure 5:**
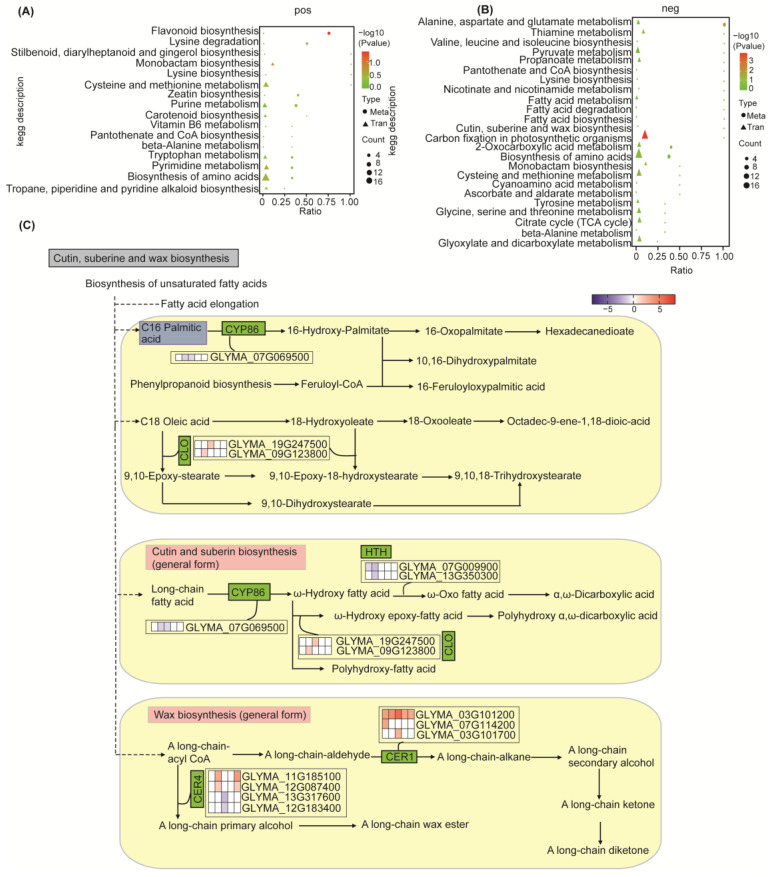
Integrated analysis of differentially expressed genes and metabolites in soybean under salt stress. (**A**,**B**) KEGG metabolic pathways for combined DEGs and DEMs identification. The abscissa is the ratio of the number of DEMs or DEGs enriched in the pathway to the number of metabolites or genes annotated in the pathway, and the ordinate is the KEGG pathway to which the metabolome and transcriptome are enriched. Count: The number of metabolites or genes enriched in the pathway. The color of the dot represents the *p*-value of the hypergeometric test, and the smaller the value, the greater the reliability and the more statistically significant the test. (**C**) The transcriptome and metabolome identified changes in Cutin, suberine, and wax biosynthesis. Gradient colors indicate log_2_-fold changes (FC) in gene expression in leaves at different time points (2, 4, 12, 24, and 48 h) compared to controls (0 h).

All the obtained differentially expressed genes and differentially expressed metabolites were subjected to pathway analysis using the KEGG database to uncover the underlying biochemical and signal-transduction pathways. Notably, we observed a significant enrichment of positive ion metabolites, leading to the identification of 16 significantly enriched metabolic pathways, mainly including flavonoid biosynthesis, amino acid biosynthesis and degradation, pantothenic acid and coenzyme A biosynthesis, and pyrimidine and purine metabolism (Figure 5A). Furthermore, we also detected a significant enrichment of negative ion metabolites, which allowed us to identify 24 differential metabolic pathways (Figure 5B). These pathways were mainly associated with fatty acid biosynthesis, degradation, and metabolism; cutin, suberine, and wax biosynthesis; pyruvate metabolism; amino acid biosynthesis and metabolism; and the citrate cycle (TCA cycle).

##### Response of Cuticle Metabolism After Salt Stress

The cuticle is an important hydrophobic protective film in the above-ground part of plants, which is of great significance for plants to adapt to complex and changing environments. We performed a further analysis of genes and metabolites associated with the cutin, suberine, and wax biosynthesis (Figure 5C). The transcriptome results showed that a total of 13 genes related to this metabolic pathway were identified to change significantly after salt stress, of which seven DEGs were upregulated and six DEGs were downregulated. *Eceriferum* (*CER*) is an important gene family that plays a key role in the elongation of ultra-long-chain fatty acids and the biosynthesis of epidermal wax under biotic and abiotic stress conditions. Our results identified a total of three *CER1* families and four *CER4* families. Specifically, the three genes within the *CER1* family were significantly upregulated after salt stress. For instance, GLYMA_03G101700 was upregulated by sixfold after 12 h of salt stress, and GLYMA_07G114200 was upregulated by ninefold after 2 h of salt stress. Moreover, GLYMA_03G101200 increased more than eight times during five time periods after salt stress, with its relative content rising by 51 times in 12 h after salt stress. However, although the CER1 protein is an aldehyde decarbonylase, its exact molecular function remains to be determined.

Regarding the *CER4* family, two out of the four identified genes were upregulated (GLYMA_12G087400 and GLYMA_11G185100), while the other two were downregulated (GLYMA_13G317600 and GLYMA_12G183400). The *CER4* family gene encodes an alcohol-forming fatty acyl-CoA reductase that participates in epidermal wax biosynthesis. Cell lines carrying recessive mutations lack primary alcohol and display a shiny stem surface.

Two genes encoding caleosins were identified, and both were elevated after salt stress, namely GLYMA_19G247500 encoding CLO2 and GLYMA_09G123800 encoding CLO6. Meanwhile, two genes of the identified hemoglobin P450 family decreased after salt stress. In addition, the relative abundance of genes (GLYMA_07G009900 and GLYMA_13G350300) of the two *HOTHEAD* families decreased after salt stress. The *HOTHEAD* gene is a gene required to limit cell-to-cell interactions between epidermal cells during flower development. Our metabolic results showed that palmitic acid, which is involved in the biosynthesis pathways of cutin, suberine, and wax, was significantly downregulated after salt stress. In conclusion, our results suggest that plant cuticles are regulated in response to salt stress.

#### 2.5.2. Integrative Analysis of Proteomic and Transcriptomic Data

**Figure 6:**
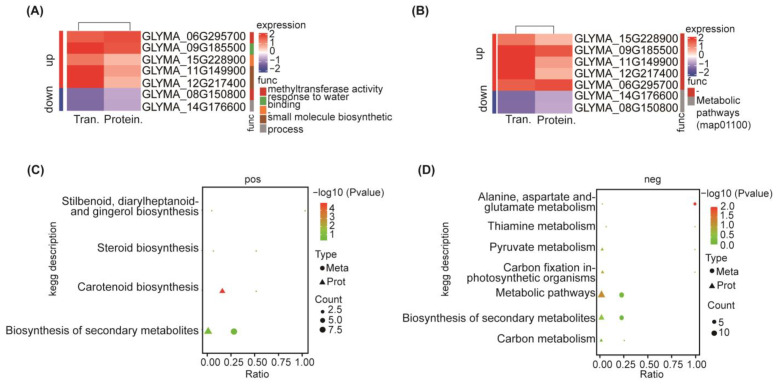
Integrated analysis of differentially expressed genes, proteins, and metabolites in soybean under salt stress. (**A**,**B**) DEGs and DEPs combined analysis of enriched GO function and KEGG metabolic pathway. The red color represents upregulation and the blue color represents downregulation, and the horizontal clustering is a clustering of expressions at the proteome and transcriptome levels, i.e., the expression patterns of proteins and genes in a cluster are similar. The gradient color indicates the log_2_ (FC), Count: the number of proteins and genes enriched in the pathway after stress compared to the control group. (**C**,**D**) KEGG metabolic pathways for combined DEPs and DEMs identification. The abscissa is the ratio of the number of DEMs or DEPs enriched in the pathway to the number of metabolites or proteins annotated in the pathway, and the ordinate is the KEGG pathway to which the metabolome and proteome are enriched. Count: The number of metabolites or proteins enriched in the pathway. The color of the dot represents the *p*-value value of the hypergeometric test, and the smaller the value, the greater the reliability and the more statistically significant the test.

The difference in fold change between the genes (proteins) identified by the transcriptome and the proteome in the two omics was analyzed for correlation (Figure S5C). By conducting a comprehensive analysis of the proteomic and transcriptomic data, we identified seven pairs of genes and proteins that exhibited significant differences in both omics. Notable observations include two pairs demonstrating methyltransferase activity, another pair associated with the GO function of small-molecule biosynthesis process, another pair linked to the GO function of binding, and a further pair related to the GO function of response to water. Additionally, there are two pairs for which GO function has not been identified (Figure 6A). Furthermore, two pairs exhibited enriched KEGG pathways in metabolic pathways (Figure 6B). Following 24 h of salt stress, the genes and proteins of these two pairs were downregulated, while the other pairs showed upregulation (Table S21).

#### 2.5.3. Integrative Analysis of Metabolomic and Proteomic Data

Correlation analysis was performed based on the Pearson correlation coefficient between DEPs and DEMs (Tables S22 and S23). A correlation coefficient less than 0 indicates a negative correlation, while a coefficient greater than 0 indicates a positive correlation. Using a correlation coefficient absolute value greater than or equal to 0.95 and a *p*-value ≤ 0.01 from the joint analysis, we identified a total of 44 negative DEMs and 82 positive DEMs that were positively correlated with DEPs. Additionally, 67 positive DEMs and 39 negative DEMs were negatively correlated with DEPs. The DEP with the highest number of positive correlations with positive DEMs was G0Z350, which is annotated as zeaxanthin epoxidase, a chloroplastic protein. In contrast, the DEP with the highest number of negative correlations with positive DEMs were I1M5Q4, an ADP, ATP carrier protein. The most abundant DEP positively correlated with negative DEMs are I1JIH2, an uncharacterized protein whose function remains to be studied. Interestingly, I1M5Q4 (ADP, ATP carrier protein) is the protein negatively associated with the largest number of negative ion DEMs.

All the obtained differentially expressed proteins and differentially expressed metabolites were mapped to the KEGG pathway database to identify their associated biochemical and signal-transduction pathways. The positive ion metabolite results were enriched in four main differential metabolic pathways: stilbethylene, diaryl heptane, and gingerol biosynthesis, steroid biosynthesis, carotenoid biosynthesis, and secondary metabolite synthesis (Figure 6C). On the other hand, the negative ion metabolite results were enriched in seven metabolic pathways, including alanine, aspartic acid, and glutamate metabolism, pyruvate metabolism, carbon fixation in photosynthetic organisms, thiamine metabolism, metabolic pathways, biosynthesis of secondary metabolites, and carbon metabolism (Figure 6D).

### 2.6. The Integrated Analysis of the Transcriptome, Proteome, and Metabolome in Soybean Under Salt Stress

**Figure 7:**
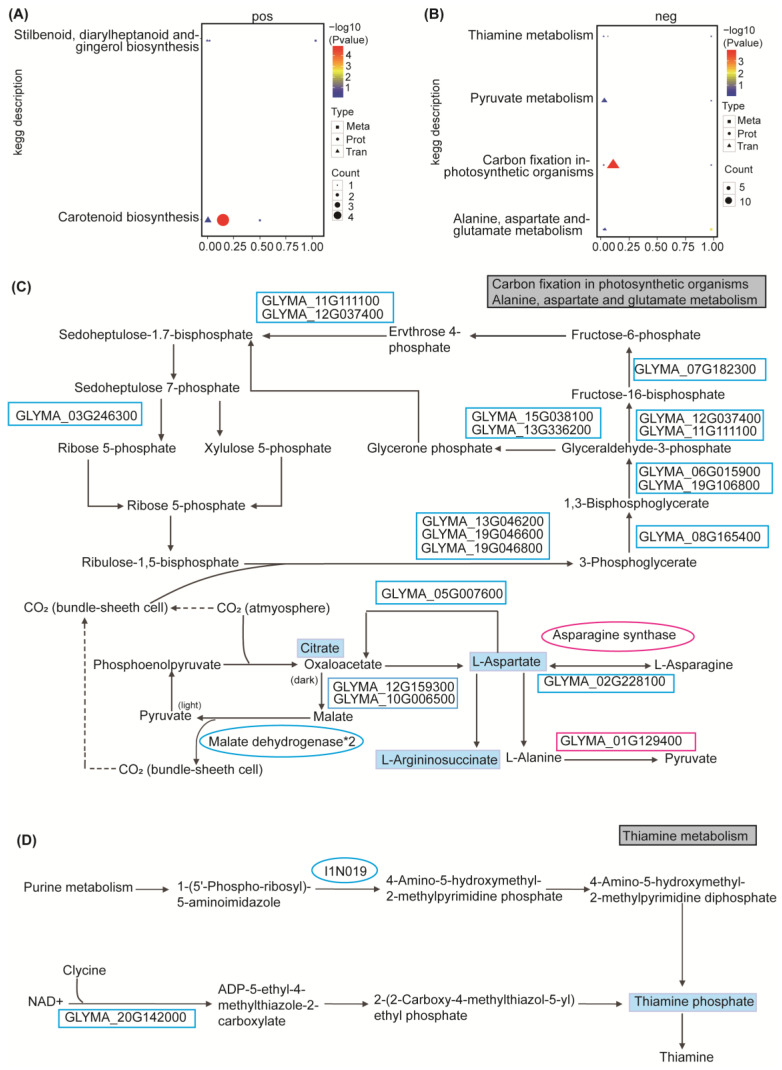
Integrated analysis of differentially expressed genes, proteins, and metabolites in soybean under salt stress. (**A**,**B**) KEGG metabolic pathways identified by integrated analysis of DEGs, DEPs, and DEMs (pos, (**A**); neg, (**B**)). (**C**,**D**) Multiomics analysis identified changes in the carbon-assimilation pathway and amino acids metabolism in photosynthetic organisms (**C**) and thiamine metabolism pathway (**D**). Boxes indicate genes, round boxes indicate proteins, and underlined boxes are metabolites. Blue color indicates downregulation, and red color indicates upregulation.

In order to provide a comprehensive understanding of biological systems, we conducted a multiomics association analysis. Our study employed a comparative approach across transcriptome, proteome, and metabolome, shedding light on the post-transcriptional regulatory status of gene expression. We established a statistical and functional correlation-based framework for integrating transcriptomic, proteomic, and metabolomic data sets (Figure S5D,E). By mapping the differentially expressed genes, proteins, and metabolites to the KEGG pathway database, we identified six major biochemical pathways and signal-transduction pathways that were implicated in the triomics analysis. These pathways include stilbenoid, diarylheptanoid, and gingerol biosynthesis; carotenoid biosynthesis; carbon fixation in photosynthetic organisms; alanine, aspartic acid, and glutamate metabolism; thiamine metabolism; and pyruvate metabolism (Table S24; Figure 7A,B).

For carbon fixation, a total of 14 DEGs, two DEPs, and one DEM were identified, all showing a decrease in relative contents after exposure to salt stress. In the alanine, aspartic acid, and glutamate metabolic pathways, three DEGs, one DEP, and three DEMs were identified, with most exhibiting downregulation. Notably, protein I1LLM2 and gene GLYMA_01G129400 were exceptions, being upregulated (Figure 7C). GLYMA_01G129400 encodes alanine: glyoxylicate aminotransferase, and its relative content begins to accumulate 1 h after the onset of salt stress, promoting pyruvate production. After 24 h of salt stress, the relative content of protein I1LLM increased by 1.5-fold (Table S10), representing asparagine synthetase.

In the thiamine metabolism pathway, 1 DEG, 1 DEP, and 1 DEM was identified, respectively (Figure 7D). Specifically, gene GLYMA_20G142000, which belongs to the *THI4* family, plays a role in the production of thiazole moiety of thiamine, serving dual functions in thiamine biosynthesis and mitochondrial DNA damage tolerance. The protein I1N019 is a ThiC-related domain-containing protein involved in thiamine synthesis. Thiamine phosphate further contributes to the overall thiamine synthesis process. Interestingly, after 24 h of salt stress, the relative content of the three omics in this pathway decreased.

One DEG, one DEP, and one DEM were identified in the stilbenoid, diarylheptanoid, and gingerol biosynthesis pathway (Figure S6A). The gene GLYMA_07G021600 exhibited increased expression after 24 h of salt stress, encoding hydroxycinnamic-CoA shikimulate/quinic acid hydroxycinnamyltransferase (HCT). This enzyme is involved in the synthesis and breakdown of hydroxycinnamic esters related to the phenylpropanoid pathway (coumaryl/caffeoylshikimic and quinate), influencing flavonoid accumulation, thereby affecting auxin transport and plant growth. The increase in curcumin content can be attributed to the substantial production of reactive oxygen species (ROS) in soybean after salt stress, enhancing the plant’s oxidative defense capability. Protein K7M862, an O-methyltransferase family protein, catalyzes the methylation of hydroxycinnamic acid derivatives in this pathway for the production of methylated plant polyphenols, including lignin, the relative content of which decreased after 24 h of salt stress (Table S10). This pathway exhibited positive responses in all three omics data sets following soybean exposure to salt stress, making it a promising candidate for enhancing soybean salt tolerance.

Two DEGs, four DEPs, and one DEM were identified in the carotenoid metabolism pathways (Figure S6B). The upregulation of zeaxanthin cyclooxygenase and its encoding gene GLYMA_01G186200 promoted the synthesis of violaxanthin and antheraxanthin. In the pyruvate metabolism pathway, six DEGs, two DEPs, and one DEM were identified (Figure S6C). Interestingly, an uncharacterized gene was found to be upregulated in the conversion of phosphoenolpyruvate to pyruvate.

### 2.7. The Results of DEGs Analysis Were Verified by RT-qPCR

**Figure 8:**
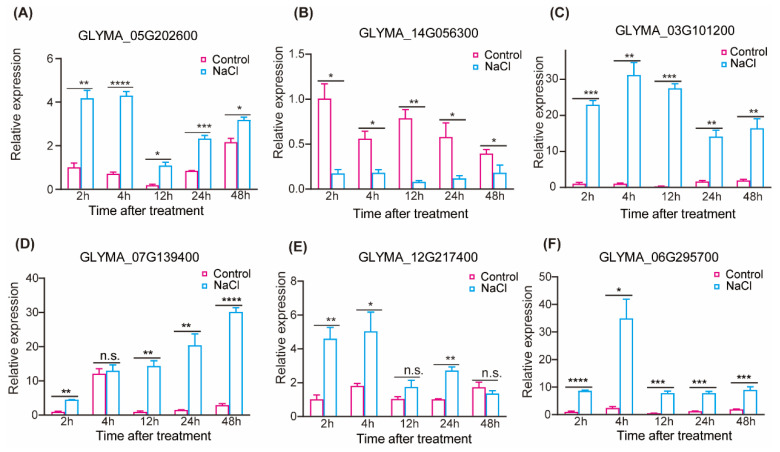
RT-qPCR validation of DEGs. (**A**–**F**) RT-qPCR validation of DEGs (**A**)**.** GLYMA_05G202600; (**B**). GLYMA_14G056300; (**C**). GLYMA_03G101200; (**D**). GLYMA_07G139400; (**E**). GLYMA_12G217400; (**F**). GLYMA_06G295700. The relative expression levels of genes at different stages of 300 mM NaCl treatment. Each bar represents the average ± SD, *n* = 3. (*t* test, n.s.: *p* > 0.05; *: *p* ≤ 0.05; **: *p* ≤ 0.01; ***: *p* ≤ 0.005; ****: *p* ≤ 0.001).

To validate the multiomics data, we performed qRT-PCR validation on 12 DEGs selected from various time points of the transcriptome and integrated omics data. Seven DEGs were chosen based on different time periods in the transcriptome data: GLYMA_05G202600, GLYMA_11G050900, GLYMA_17G036400, GLYMA_14G056300, GLYMA_13G306900, GLYMA_15G250100, and GLYMA_16G012000. From the combined transcriptome and proteome analysis, we selected four DEGs: GLYMA_07G139400, GLYMA_12G217400, GLYMA_08G150800, and GLYMA_06G295700. Additionally, GLYMA_03G101200 was chosen for the combined transcriptome and metabolome analysis. The results consistently demonstrated that 10 DEGs were upregulated and two DEGs were downregulated, providing further validation of the multiomics data using RT-qPCR (Figure 8A–F; Figure S7). The gene primers used for RT-qPCR are listed in Table S25. The results of melting curves analysis of the amplicons can demonstrate the absence of additional non-specific PCR amplicons (Figure S8).

## 3. Discussion

**Figure 9:**
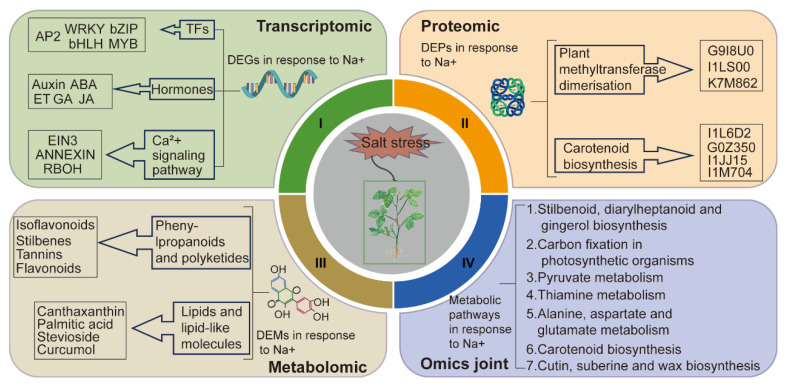
Omics analysis of the regulatory mechanism after salt stress in soybean. The first part is the key DEGs identified in transcriptomics, the second part is the key DEPs identified in proteomics, the third part is the DEMs identified in metabolomics, and the fourth part is the significant changes identified in the KEGG metabolic pathway identified by the three omics.

With the increase of population and the continuous increase of saline–alkali area, we need to cultivate crops with adaptability to adversity and improve the utilization rate of saline–alkali land to ensure food security. Data from omics tools can improve the reliability and speed of breeding programs and develop genotypes with adversity-adaptive to ensure food security [54]. Through evolutionary development, plants have developed active defense mechanisms to resist stress [55].Our research reveals the molecular mechanisms underlying soybean’s response to salt stress through a multiomics approach (Figure 9). Transcriptomic analysis revealed significant changes in transcription factors, hormone-related groups, and calcium ion signaling. Joint two-omics analyses identified a number of metabolic pathways that were significantly altered after salt stress, including cutin, suberine, and wax biosynthesis, flavonoid biosynthesis, and steroid biosynthesis. The triomics analysis further indicated that six pathways exhibited significant changes, including stilbenoid, diarylheptanoid, and gingerol biosynthesis; carotenoid biosynthesis; carbon fixation in photosynthetic organisms; alanine, aspartate, and glutamate metabolism; thiamine metabolism; and pyruvate metabolism. These findings provide potential targets and a theoretical basis for cultivating stress-tolerant plant varieties.

### 3.1. Plants Respond to Salt Stress Through Anabolites

Plants stabilize cell and protein structures by synthesizing compatible osmotic substances that reduce the osmotic potential within cells [56,57,58], thus responding to osmotic stress due to salt stresses such as proline, soluble protein, betaine, sugars (sucrose, fructose), polyols (mannitol, sorbitol), and polyamines [59,60,61,62]. Our results showed that many genes related to sugar synthesis were significantly enriched after salt stress, including the synthesis of raffinose and trehalose. GLYMA_05G003900 encodes raffinosid synthase, which has the potential to enhance drought resistance. The role of this gene in salt tolerance could be further explored. At present, the stress resistance of trehalose phosphate synthase gene has been studied in rice [63]; this provides ideas for our future research. Metabolomic results also demonstrated the accumulation of stevioside and fructose, and the accumulation of polyols (Pantothenic acid and Sequoyitol) after salt stress. These findings are consistent with previous findings [64]. However, most of the previous studies on the stress resistance of polyols have focused on mannitol and sorbitol [65], and the sequoyitol found in this result has not been reported in depth.

Plants may also produce substances with antioxidant properties to maintain redox balance. Our results showed the accumulation of 16 flavonoid metabolites, among which the content of catechins increased 13-fold after salt stress. Flavonoids are important metabolites in response to abiotic stress, which accumulate in response to ROS production when plants are subjected to salt stress. Moreover, it has been demonstrated that the application of exogenous catechins under salt stress enhances the antioxidant defense capacity of pepper (*Capsicum annuum* L.) [66]. In addition, the results of this study showed that praeruptorin A was also accumulated, and previous studies [67] showed that its accumulation was positively correlated with phenolic and flavonoid content, as well as SOD and POD activity, suggesting that this metabolite may have antioxidant capacity.

### 3.2. The Role of Plant Hormones in Abiotic Stress

Plant hormones are involved in plant response to salt stress through complex signaling pathways by integrating various developmental and environmental signals. In this study, a total of 106 plant hormone-related genes were identified, which changed significantly after salt stress.

ABA is the most important “anti-stress hormone” in plants [68]. ABA accumulates under salt stress and is involved in the osmosis of salt stress and the regulation of Ca^2+^ and ROS [69,70]. Our metabolic results also identified the accumulation of ABA. ABA binds to the receptor PYR1/PYLS/RCARs protein to form a complex, binds to and inhibits PP2C, and releases SnRK2 [71]. Activated SnRK2 phosphorylates downstream substrates, initiating ABA signaling pathways and stress response processes [72]. The results of this study showed that all four coding genes of this protein family were downregulated. AFPs can respond to abiotic stresses such as drought and high salinity through transcriptional induction [73]. The results of this study showed that all five genes of AFP3 were upregulated. ABA-dependent pass-induces the binding of bZIP transcription factor (TFs) to ABA response element (ABRE) and upregulates RD29B [74]. Our results showed that both genes for RD29B were upregulated.

Gibberellin (GA) is regulated in response to salt stress at different growth stages of plants. Accumulation induces seed germination, and a decrease in gibberellin levels or gibberellin signaling after germination necessitates improved plant tolerance to salt stress. The results of this experiment showed that the three genes of gibberellin receptor were upregulated after salt stress. The results of this study showed that all three genes of gibberellin receptor were upregulated after salt stress, and previous studies showed that the homologous genes in their rice interacted with DELLA protein in vivo in the presence of GA4 [75,76]. DELLA protein SLR1 is an inhibitor of GA signaling that protects against salt stress by inhibiting plant growth [77]. Therefore, we speculate that soybeans may also be able to protect against salt stress in this way.

ET accumulates after salt stress and has been shown to play an important role in stress [78,79]. In this study, all ET-related genes were upregulated after salt stress, indicating that ethylene signal positively regulated the salt tolerance of plants. The results were enriched for three *EIN* genes, and previous transcriptome analysis showed that salt induced the expression of many *EIN3/eil1*-dependent defense-related genes [80], which is consistent with our results. Expression levels of the direct target genes *ESE1* and *ERF1* (Ethylene response factors1) of Ethylene insensitive 3 (*EIN3*) were also significantly increased after NaCl treatment [80,81]. Our transcript results do not show this phenomenon.

In addition, transcriptome results showed that all nine JA-related genes were upregulated after salt stress, and only one gene was involved in JA biosynthesis, six genes were involved in JA-mediated signaling pathway, and two genes played a role in JA metabolism. Previous studies have shown that JA levels are elevated under salt stress and JA signaling is activated [82,83]. Reducing the production or accumulation of JA leads to high sensitivity to salt in tomato [84] and rice [85], while JA biosynthesis improves salt tolerance in wheat and Arabidopsis [86]; this is consistent with our results. Eight of the 43 auxin-related genes were downregulated, while the rest were upregulated. In conclusion, after salt stress, not only is there synthesis of plant hormones in response to salt stress, but also the activation of plant hormone-mediated signaling pathways to resist stress.

### 3.3. Changes in Transcription Factors After Salt Stress

Transcription factor (TF) is a protein molecule with a special structure that can regulate gene expression and plays an important regulatory role in plant growth and development and stress-defense response. They receive upstream signals and regulate the expression of downstream-related resistance genes by binding to the corresponding cis-regulatory sequences. At present, many transcription factors have been identified to be related to salt stress, mainly WRKY, MYB, bHLH, AP2/ERF (apetala2/ethylene response factor), bZIP (Basic leucine zipper), and other transcription factor families.

WRKY transcription factors can play both positive and negative regulatory functions in plants in response to salt stress [87,88]. The WRKY family we identified was upregulated after salt stress. Current studies have shown that the R2R3 subclass of MYB is the most abundant type of MYB, which is widely involved in plant hormone response, secondary metabolism, environmental stress, and other processes [89,90,91]. In this study, eight genes were identified as R2R3 subclass, among which *GmMYB111* was upregulated by six times, and the results showed that *AtMYB111* was involved in the synthesis of flavonoids in plants, and positively regulated the salt tolerance of plants [92].

AP2/EREBP transcription factors are a plant-specific superfamily of transcription factors with a conserved domain AP2/ERF that is involved in regulating plant growth, development, and response to abiotic stresses [93]. Our results showed that 16 genes with significant changes in this family after salt stress were ethylene reactive factors (ERFs) and 15 were DREB, and only four transcription factors were found in other categories. Therefore, it can be seen that the two subfamilies of ERF and DREB are mainly involved in the response to plant salt stress, which is consistent with the existing research [94]. At present, many studies have shown that *DREB* and *ERF*-related genes were induced under salt stress and improved the salt tolerance of Arabidopsis [95], rice [96], and other plants. However, there are still many genes that accumulate after salt stress in our results, and their specific functions have not been studied and verified.

### 3.4. Activation of Calcium Ion Signaling After Salt Stress

Calcium ion signals can be sensed by multiple modules and activate downstream signaling pathways to activate metabolic pathways, so as to cope with the damage caused by salt stress to plants. After salt stress, the increase of calcium concentration stimulates the production of Ca^2+^ signaling. Subsequently, these Ca^2+^ signals are sensed by various Ca^2+^ binding modules, including CAM, CDPKs [97]. Our results show a general accumulation of calcium-bound annexin. Previous studies have shown that SOS3/SCaBP8 activates the SOS2-forming complex and interacts with the Ca^2+^-dependent membrane-bound protein Annexin4 (ANN4) to form the complex SCaBP8-AtANN4-SOS2, which regulates Ca^2+^ signaling under salt stress and improves the Na^+^/H^+^ transport activity of the plasma membrane [98]. These results indicated that salt-induced Ca^2+^ signaling could activate the SOS pathway and enhance plant salt tolerance.

CDPKs play a role in multiple plant signaling pathways downstream of elevated Ca^2+^ concentrations, thereby regulating various physiological responses. CDPKs mainly alleviate salt stress by triggering the expression of several antioxidant genes and limiting the expression of NADPH oxidase to regulate stomatal conductance, ion channel-related gene expression, and ROS balance [99]. The GLYMA_05G213200 we identified encodes a calcium-dependent protein kinase 3 (CDPK3, known as CPK3 in Arabidopsis) that is expressed in both guard and mesophyll cells and plays a role in guard cell ion channel regulation. CPK3 loss-of-function mutants all exhibited a salt-sensitive phenotype, and CPK regulated the K^+^/Na^+^ ratio under salt stress [100]. The results of this study identified four CDPKs that were upregulated.

In plants, “respiratory burst oxidase homologous” (RBOH) proteins localize to the plasma membrane and have been reported to regulate various biological processes, including pathogen responses and abiotic stress tolerance, through the ability of ROS to regulate production as a second messenger [101,102]. In this study, the three genes encoding RBOH and their homologs were upregulated, but their effects and responses to salt stress need to be further studied.

### 3.5. Changes in DNA Methylation State Induced by Salt Stress

DNA methylation, an epigenetic modification, involves the addition of a methyl group primarily to cytosine residues without altering the DNA double helix sequence. This process regulates the expression of responsive genes in plant abiotic stress, thereby influencing plant response to salt stress [103]. In our study, the protein K7M862, which was screened through proteomics and transcriptomics, was downregulated after salt stress, demonstrated methyltransferase activity, and was able to catalyze pterostilbene biosynthesis from resveratrol. The transcriptome results identified four DEGs with GO functions related to DNA methylation. After salt stress, the relative content of these DEGs decreased. Some studies have shown that the level of DNA methylation in plants under salt stress tends to be enhanced [104,105], and some studies have found that the content of DNA methyltransferase-related genes in plants may be upregulated, downregulated, or unchanged after salt stress [106]. The results of this study showed that the relevant genes and proteins were mostly downregulated, and an uncharacterized protein containing a methyltransferase domain (I1LS00) was upregulated. At present, the relationship between DNA methylation and salt stress has been studied in cotton (*Gossypium hirsutum* L.) [104], sugar beet (*Beta vulgaris* L.) [107], and Arabidopsis [108]. However, how DNA methylation regulates soybean response to salt stress remains to be studied.

### 3.6. The Role of the Cuticle in Abiotic Stress

The cuticle constitutes an important hydrophobic protective layer in the aerial parts of plants. It serves as the first line of defense against pathogen invasion and also protects the plant from many abiotic stresses. The main function of cuticle is to reduce non-stomatal water loss, and it plays an important role in enhancing the resistance of plants to abiotic stresses such as drought, salt, and high temperature [109]. In this study, a total of 13 genes related to this pathway were identified to change significantly after salt stress, including three *CER1* families and four *CER4* families, which belonged to epidermal wax biosynthesis genes and were mostly upregulated after salt stress. The content of wax increased after salt stress, and the key *CER1* gene for its synthesis was upregulated after salt stress [110], which was consistent with our results. *CER4* is involved in the synthesis of very long-chain fatty acids (VLCFAs), and its family is downregulated after salt stress [111], and the results of this study show that two of the four genes of *CER4* are upregulated and two are downregulated. In addition, there were five genes related to keratin and lignin synthesis pathways, namely *Caleosins* family (*CLO2*, *CLO6*), two *HOTHEAD* genes, and *CYP* family (*CYP77B1* and *CYP86A8*), among which only the *CLO* family genes were upregulated, which was consistent with the previous research results that *CLO06* had a positive regulatory effect on salt tolerance in cotton [112]. Our results show that five of the seven upregulated genes are wax synthesis genes in the stratum corneum, so we suspect that during the time we studied, soybeans mainly relied on wax synthesis in the stratum corneum to increase the plant’s defenses.

## 4. Materials and Methods

### 4.1. Plant Materials

The cultivated soybean seeds, William 82, were dried in a vacuum oven (37 °C) for 48 h, then sown in pre-filled pots with infiltrated soil and germinated in an incubator. The incubator was set to maintain a temperature of 25 °C, relative humidity at 50%, and a light-dark cycle of 16 h and 8 h, respectively. Upon the unfolding of the first true leaf, the seedlings were divided into two groups, one exposed to 300 mM NaCl and the other to deionized water. After 2, 4, 12, 24, and 48 h of stress, the first true leaf was sampled, immediately flash-frozen in liquid nitrogen, and stored at −80 °C. Three replicates of seven leaves per replicate at each time point were used for transcriptome experiments. Additionally, the protein and metabolic groups were sampled after 24 h of salt exposure. For the proteome and metabolome experiments, samples were collected after 24 h of salt stress, with seven leaves in each of the three replicates for the proteome experiment and six replicates for the metabolome experiment. Subsequently, the samples were sent to NOVOGENE Company for testing.

### 4.2. Physiological Measurements

Use the portable photosynthetic system to repeat measurements at 0, 1, 2, 4, and 24 h following the initiation of salt stress, and determine the photosynthetic rate, transpiration rate, and stomatal conductance of soybean leaves in both control and experimental (salt-treated) groups. Randomly arrange and process the seedlings to mitigate potential positional biases.

Harvest leaves from both the control group and 6-day-old soybean seedlings, taking 15 leaves from each. Submerge the leaves in a dehydration solution (ethanol-free water: ice acetic acid = 3: 1) to achieve leaf transparency, allowing them to soak for 30 min. Once the leaves are fully transparent, remove the decolorization solution and immerse them in Basic Solution for 30 min. After discarding the Basic Solution, sequentially immerse the leaves in 60% ethanol, 40% ethanol, 20% ethanol, and 10% ethanol for 20 min each. Finally, 10% ethanol was sucked, 5% ethanol and 25% glycerin were added, and then a microscope was used for observation and image capture. Stomata number and other cell number counts were performed using Fiji software (https://fiji.sc, accessed on 16 April 2023) [113], and then the stomatal index was calculated. The stomatal index (SI) was calculated as: SI = (number of stomata)/(number of stomata + number of other epidermal cells) × 100 [114].

### 4.3. Transcriptomics Sequencing and Analysis

In this study, leaf tissues were milled with liquid nitrogen and RNA was extracted using the RNAprep Pure Polysaccharide Polyphenol Plant Total RNA Isolation Kit (spin column type) (Tiangen, Beijing, China). Total RNA was used as input material for the RNA sample preparations. Briefly, mRNA was purified from total RNA using poly-T oligo-attached magnetic beads. Fragmentation was carried out using divalent cations under elevated temperature in First Strand Synthesis Reaction Buffer (5×). First strand cDNA was synthesized using random hexamer primer and M-MuLV Reverse Transcriptase (RNase H-). Second strand cDNA synthesis was subsequently performed using DNA Polymerase I and RNase H. Remaining overhangs were converted into blunt ends via exonuclease/polymerase activities. After adenylation of 3′ ends of DNA fragments, Adaptor with hairpin loop structure were ligated to prepare for hybridization. In order to select cDNA fragments of preferentially 370~420 bp in length, the library fragments were purified with AMPure XP system (Beckman Coulter, Brea, CA, USA). Then, PCR was performed with Phusion High-Fidelity DNA polymerase, Universal PCR primers, and Index (X) Primer. At last, PCR products were purified (AMPure XP system) [115] and library quality was assessed on the Agilent Bioanalyzer 2100 system. After library validation, sequencing was performed using the illumina NovaSeq 6000 (Illumina, San Diego, CA, USA), S4 Suite assembly. The quantitative analysis utilized the featureCounts tool (version: 1.5.0-p3) [116] in the subread software. Upon completion of gene expression quantification, statistical analysis was conducted, incorporating original readcount normalization, mainly focusing on the correction of sequencing depth. The statistical model calculated the probability for hypothesis testing (*p*-value), ultimately correcting for multiple hypothesis tests to obtain the false discovery rate (FDR) value (often represented as padj). We applied criteria (|log_2_(FC)| >= 1 & padj <= 0.05) to screen for differentially expressed genes (DEGs). Finally, the functional analysis of the selected DEGs was carried out.

### 4.4. Proteomics Sequencing and Analysis

The sample was ground individually in liquid nitrogen and lysed with SDT lysis buffer (containing 100 mM Nacl) and 1/100 volume of DTT, followed by 5 min of ultrasonication on ice. After reacting at 95 °C for 8–15 min and ice-bath for 2 min, the lysate was centrifuged at 12,000× *g* for 15 min at 4 °C. The supernatant was taken and added with sufficient IAM to react for 1 h at room temperature in the dark. Then, samples were completely mixed with four times volume of precooled acetone by vortexing and incubated at −20 °C for at least 2 h. Samples were then centrifuged at 12,000× *g* for 15 min at 4 °C, and the precipitation was collected. After washing with 1 mL cold acetone, the pellet was completely dissolved by Dissolution Buffer (DB buffer) [117,118]. 

This experiment used Tandem Mass Tags (TMT) technology by Thermo SCIENTIFIC (Waltham, MA, USA) for quantitative proteomics analysis [119]. Proteome Discoverer software was used to analyze the data from mass spectrometry detection and obtain protein-quantification results. Protein-difference analysis began by selecting the sample pairs for comparison. The ratio of the mean quantification values from all biological replicates for each protein in the comparison sample pair was used to determine the fold change (FC). T-tests were then conducted on the relative quantification values of each protein in the two comparison samples, generating corresponding *p*-values. DEPs were screened based on a fold change threshold of 1.5 (upregulated by more than 1.5 times or downregulated by less than 0.67 times) and a *p*-value < 0.05. Finally, a series of differential protein function analysis, such as GO and KEGG function enrichment analysis and interaction network analysis, was carried out for the screened differential proteins.

The StringDB protein-protein interaction database (http://string-db.org/, accessed on 25 March 2023) was used to analyze the interaction of identified proteins. If there was a corresponding species in the database, the sequence of the corresponding species was directly extracted. If not, the sequence of the proximal species was extracted. Then, the sequences of the differential proteins were compared with the extracted sequences to obtain the corresponding interaction information and construct a network diagram.

### 4.5. Metabolomics Sequencing and Analysis

Tissues (100 mg) were individually grounded with liquid nitrogen, and the homogenate was resuspended with prechilled 80% methanol by well vortex. The samples were incubated on ice for 5 min and then were centrifuged at 15,000× *g*, 4 °C for 20 min. Some of the supernatant was diluted to final concentration containing 53% methanol by LC-MS grade water. The samples were subsequently transferred to a fresh Eppendorf tube and then were centrifuged at 15,000× *g*, 4 °C for 20 min. Finally, the supernatant was injected into the LC-MS/MS system to conduct non-targeted metabolic profiling [120,121,122]. Compound Discoverer 3.1 data processing software was used to analyze and process the data to obtain quantitative results of metabolites. The screening of differentially expressed metabolites (DEMs) mainly referred to three parameters: VIP, FC, and *p*-value. VIP refers to the PLS-DA model Variable Importance in the Project [123]. VIP value indicates the contribution of metabolites to the grouping group; The FC (Fold Change) refers to the difference between the difference, which is the ratio of the average value of all biological quantities in each metabolites in the comparison group; *p*-Value obtained [124] through T-test calculation, indicating a significant difference in differences. DEMs were screened according to the criteria of VIP > 1.0, FC > 1.5 or FC < 0.667 with *p*-value < 0.05 [123,125,126]. Finally, the identified metabolites were annotated using the KEGG database (https://www.genome.jp/kegg/pathway.html, accessed on 25 March 2023), the HMDB database (https://hmdb.ca/metabolites, accessed on 25 March 2023), and the LIPIDMaps database (http://www.lipidmaps.org/, accessed on 25 March 2023).

### 4.6. Functional Enrichment Analysis of DEGs, DEPs, and DEMs

The clusterProfiler software (https://bioconductor.org/packages/release/bioc/html/clusterProfiler.html, accessed on 25 March 2023) was used to analyze the GO function enrichment of DEGs and DEPs, and the KEGG pathway enrichment analysis of DEGs, DEPs, and DEMs was carried out. The enrichment analysis is based on the principle of hypergeometric distribution, in which the differential gene set is the DEGs, DEPs, and DEMs obtained by differential significant analysis and annotated to the GO database (http://www.geneontology.org/, accessed on 25 March 2023) or KEGG database gene set. The background gene set is the gene set for all genes, proteins, and metabolites that have been analyzed differentially and annotated into the GO or KEGG database.

### 4.7. Real-Time Quantitative PCR (RT-qPCR) Analysis mRNA Expression

RT-qPCR analysis was performed using the CFX96 RT-qPCR platform (BioRad, Hercules, CA, USA). For each RT-qPCR reaction, the following components were included: 10 μL of Hieff^®^ qPCR SYBR Green Master Mix (No Rox) (Yeasen, Shanghai, China), 8.2 μL of ddH_2_O, 1 μL of diluted cDNA, and 0.4 μL of forward/reverse primers. The reaction conditions for RT-qPCR were as follows: 95 °C for 5 min, 95 °C for 10 s, 55–60 °C for 20 s, 72 °C for 20 s, and a total of 40 cycles. Each sample was performed in triplicate, and the mean and standard deviation were calculated. *Tubulin* was used as internal controls for normalization. The fold changes (2^−∆∆Ct^) [127] were determined relative to the control sample.

## 5. Conclusions

In this study, the transcriptome, proteomic, and metabolomic responses of soybean seedlings after salt stress were comprehensively analyzed. Our findings indicate that, in response to salt stress, soybeans synthesize various metabolites that help mitigate the damage caused by such stress, with DNA methylation contributing to this process. Moreover, calcium ion signaling, plant hormone signaling, and transcription factors cooperate with one another in response to salt stress, highlighting the need for further research in this area. Additionally, the synthesis of plant cuticles is also influenced after exposure to salt stress, which may enhance the salt tolerance of soybean. Our study identified several potential targets for soybean improvement, and it is anticipated that novel salt-tolerant soybean varieties can be developed in the future through the application of overexpression, gene editing, and other technologies. In conclusion, our study offers a theoretical foundation for improving their salt tolerance.

## Data Availability

All data sets in this study have been uploaded to a public database, and the following are links to the data sets. Transcriptomics data has been deposited in the Sequence Read Archive (SRA) database with the identifier PRJNA1110806. The full data set will be here http://www.ncbi.nlm.nih.gov/bioproject/1110806 accessible. Proteomics data has been deposited in the ProteomeXchange database with the identifier PXD052320. The full data set will be accessed here https://www.ebi.ac.uk/pride/review-dataset/98b660d87ee641faa40f5f1dcc3b8e1f. Username: reviewer_pxd052320@ebi.ac.uk; Password: E8BmyTf4USaY.Metabolomics data have been deposited to the EMBL-EBI MetaboLights database (DOI: 10.1093/nar/gkad1045, PMID:37971328) with the identifier MTBLS10210. The complete data set will be accessed here https://www.ebi.ac.uk/metabolights/MTBLS10210.

## Supplementary Materials

The supporting information can be downloaded at: https://www.mdpi.com/article/10.3390/ijms252413559/s1.
