# Supplementary material for: Integrated Transcriptomic, Proteomic, and Metabolomic Analyses Revealed Molecular Mechanism for Salt Resistance in Soybean (Glycine max L.) Seedlings
Source: Int J Mol Sci. 2024 Dec 18;25(24):13559. doi: 10.3390/ijms252413559 (PMC11678865; doi:10.3390/ijms252413559)
Supplement: Supplementary file 1 [file ijms-25-13559-s001.zip › Supplementary materials-Figure.pdf]

Figure S1

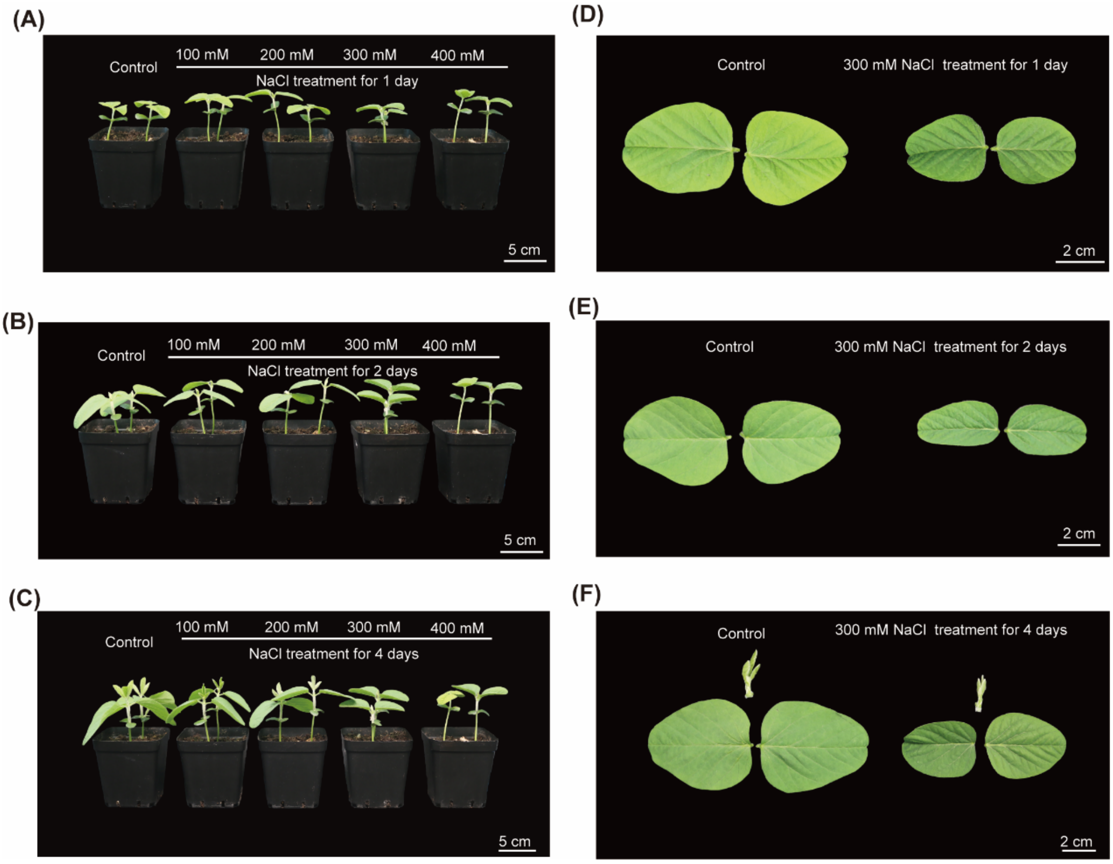

Figure S1. Phenotype of soybean seedlings under salt stress.

(A-C) Phenotypic changes of soybean seedlings under different concentrations of NaCl stress on 1d (A), 2d (B), and 4d (C).

(D-F) Phenotypic changes of soybean leaves at 1d (D), 2d (E) and 4d (F) under 300mM NaCl stress.

Figure S2

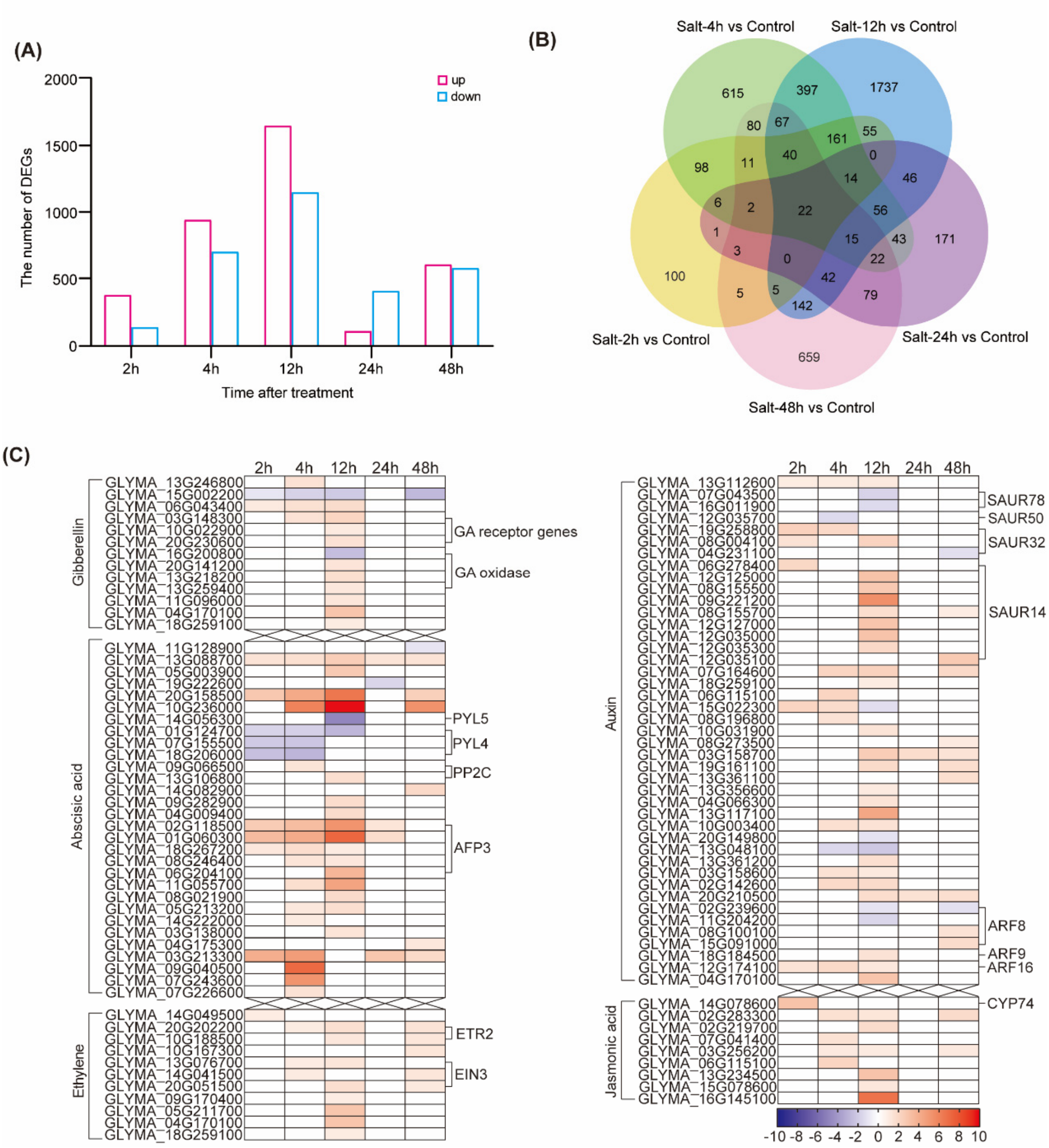

Figure S2. Transcriptome changes of soybean seedlings under salt stress.

(A) DEGs identified at different times under salt stress, and the number of genes with different differences in abscissa and ordinate at different times.

(B) Venn diagram of co-expression of DEGs at different times after salt stress, showing the number of genes uniquely expressed in each group/sample, and overlapping regions showing the number of genes co-expressed in two or more groups/samples.

(C) Changes in gene expression associated with plant hormones. Gradient colors indicate log_2-fold changes (FC) in gene expression in leaves at different time points (2, 4, 12, 24, and 48 h) compared to controls (0 h).

Figure S3

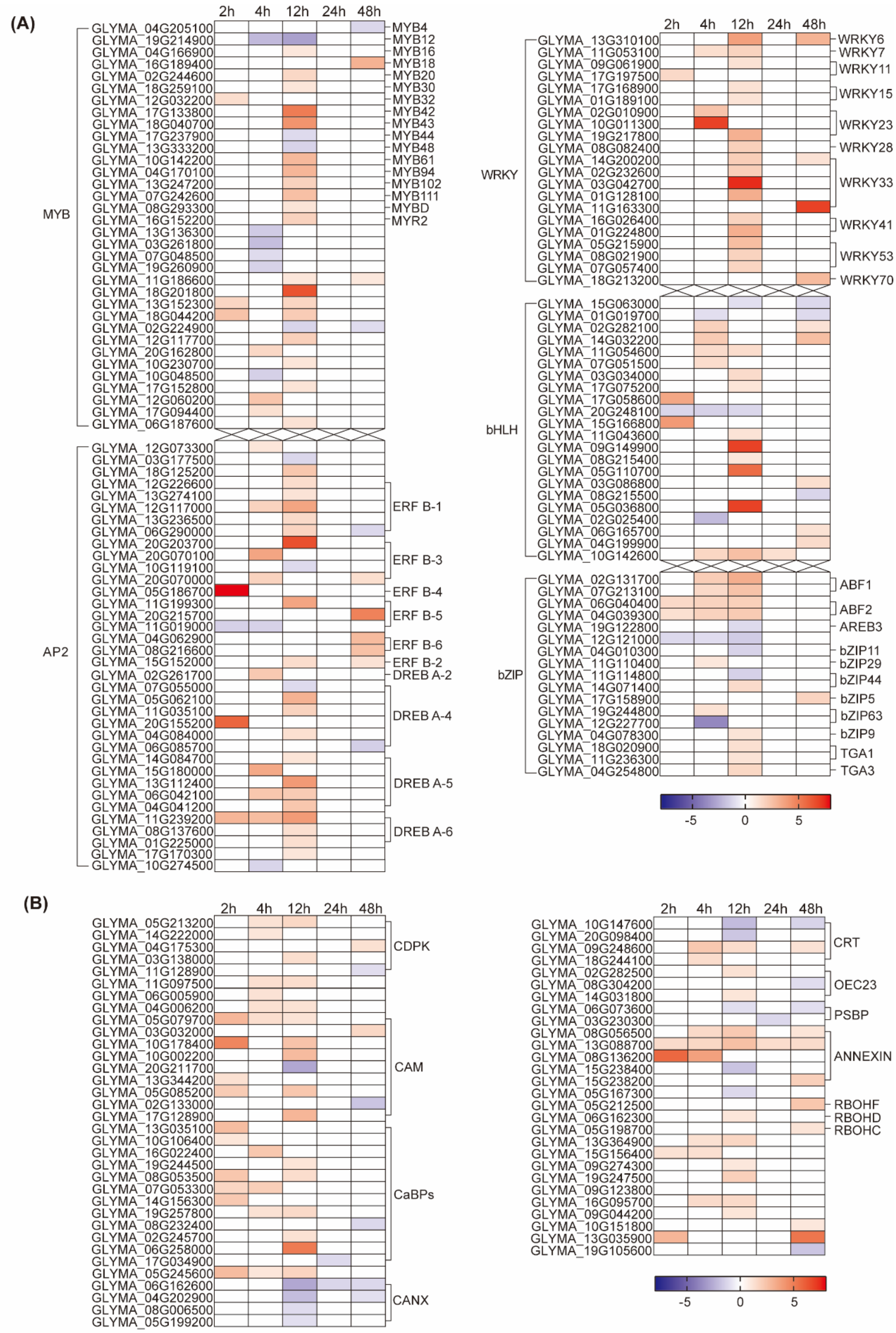

Figure S3. Changes in the expression of key genes.

(A-B) Changes in gene expression associated with transcription factors (A) and

Calcium ion signaling (B). Gradient colors indicate log_2-fold changes (FC) in gene expression in leaves at different time points (2, 4, 12, 24, and 48 h) compared to controls (0 h).

Figure S4

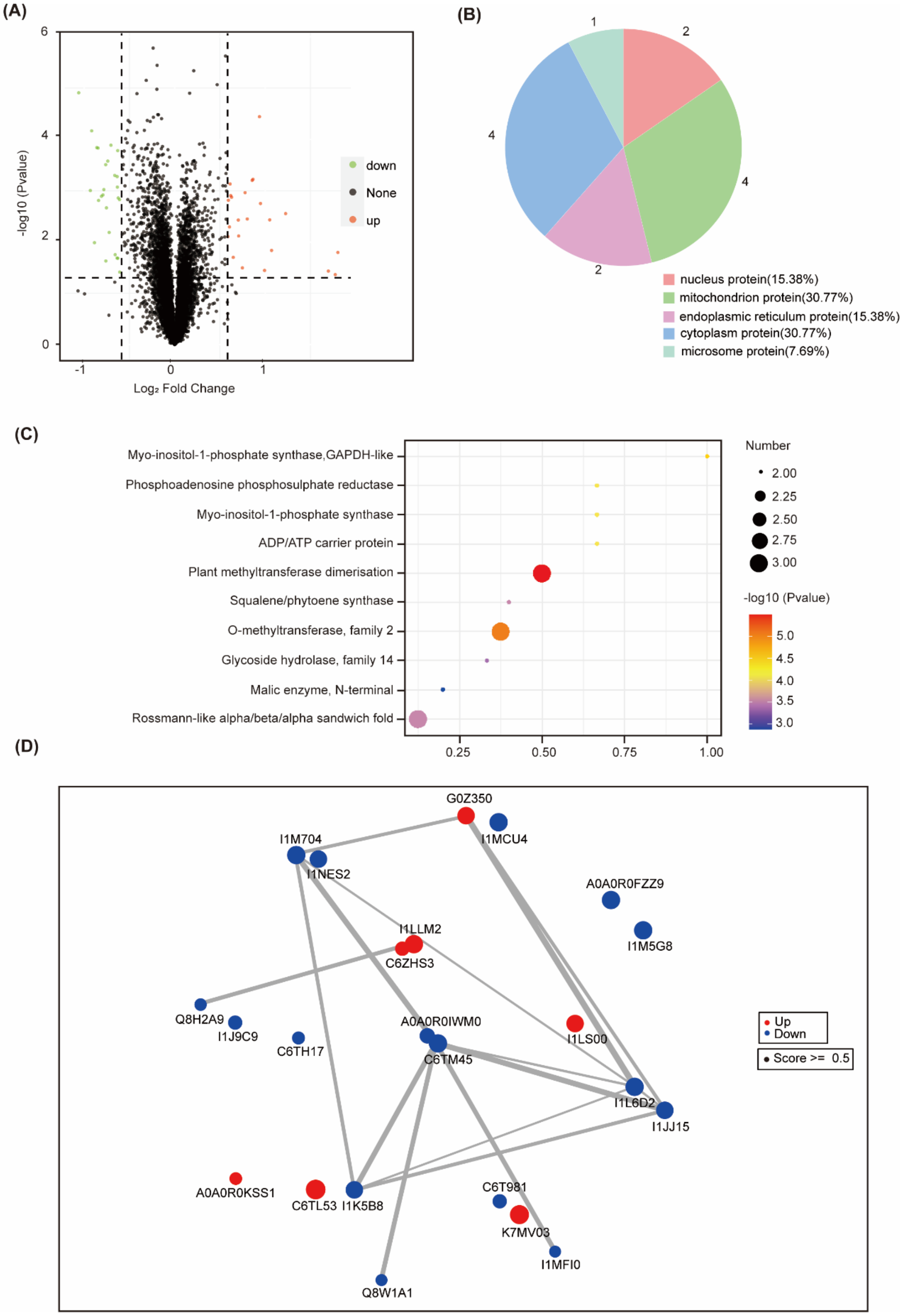

Figure S4. Proteome changes of soybean seedlings under salt stress.

(A) Diagram of DEPs volcanoes. For each protein, take the logarithm with 2 as the base, and take the P-value value as the base with 10 as the absolute value of the

logarithm. The abscissa represents the difference factor (log_2 value), the vertical axis represents the P-value ($-log_{10}$ value), black represents the protein with no significant difference, red represents the up-regulated protein, and green represents the down-regulated protein.

(B) Subcellular localization analysis of DEPs.

(C) DEPs domain enrichment. The abscissa is the ratio of the number of differential proteins in the corresponding domain to the total number of proteins identified in the domain. The color of the dot represents the P-value value of the hypergeometric test, and the redder the color, the smaller the P-value value, the greater the reliability of the test, and the more statistically significant it is. The size of the dot represents the number of differential proteins in the corresponding domain, and the larger the dot, the more differential proteins within that domain.

(D) Protein-protein interaction analysis. Each node in the interaction network represents a protein, the size of the node represents the number of proteins interacting with it, the larger the node, the more proteins interacting with it, the color of the node indicates the expression level of the protein in the comparison pair, the red color represents the protein is significantly higher expression, and the green color represents the protein is significantly lower expression.

Figure S5

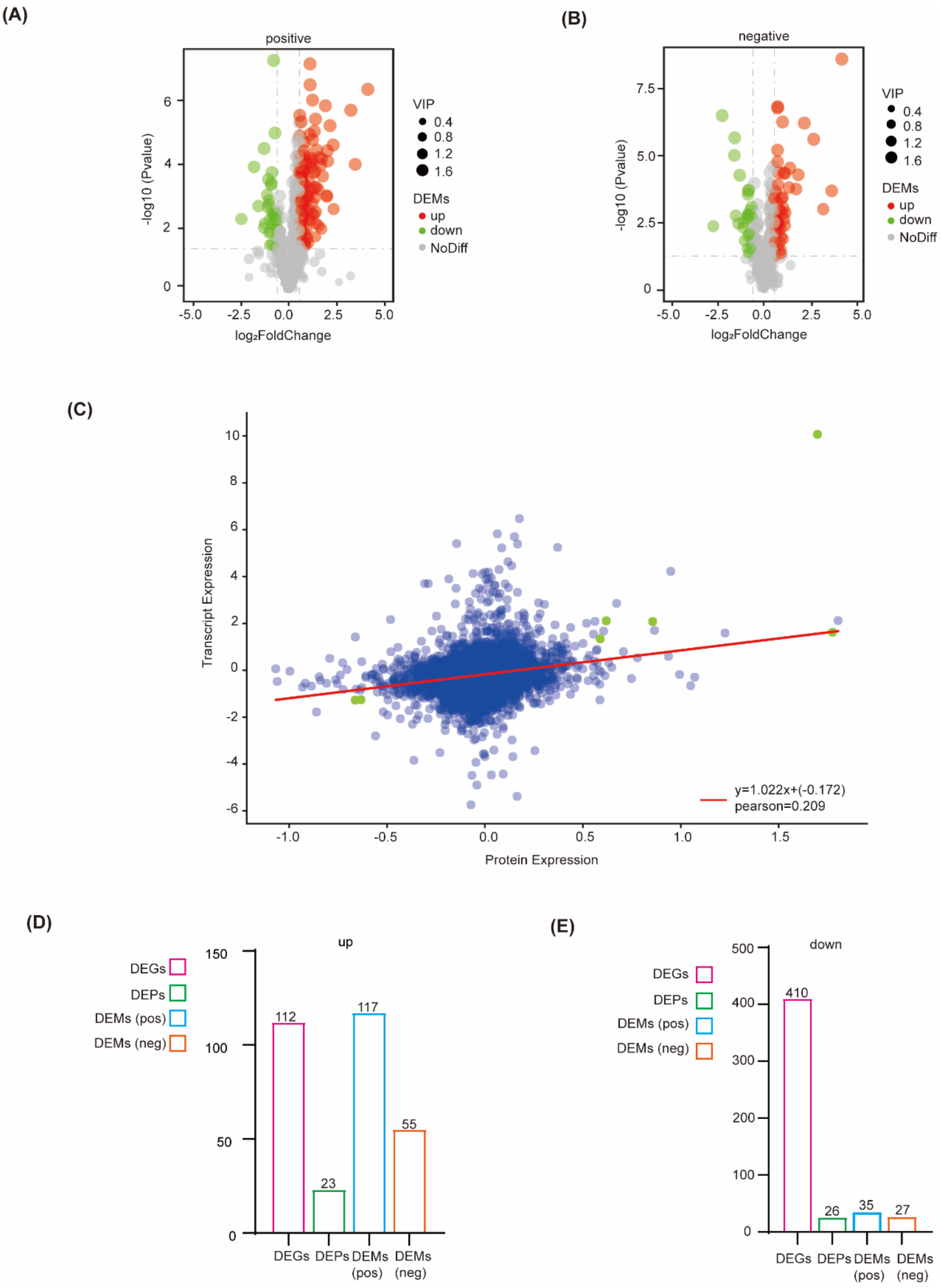

Figure S5. Integrated analysis of DEGs, DEPs and DEMs in soybean under salt stress. (A-B) Volcano diagram of positive and negative ion metabolites. The abscissa represents the fold change of metabolites in different groups ($\log_2$(Fold Change)), the ordinate represents the level of difference significance ($-\log_{10}$(P-value)), each point in

the volcano plot represents a metabolite, the metabolites that are significantly up-regulated are represented by red dots, the metabolites that are significantly down-regulated are represented by green dots, and the size of the dots represents the VIP value.

(C) Transcriptome and proteome expression correlation analysis. Each dot represents a protein, the green dot represents the protein with significant difference, the blue dot represents the protein with no significant difference, the abscissa is the fold of difference (log_2 value) of the corresponding protein in the proteome data, and the vertical axis is the fold of difference (log_2 value) of the corresponding gene in the transcriptome data.

(D-E) Statistics of up- (D) and down- (E) regulation of DEG, DEP and DEM (positive and negative) in soybean after 24 hours of salt stress. The vertical axis represents the quantity.

Figure S6

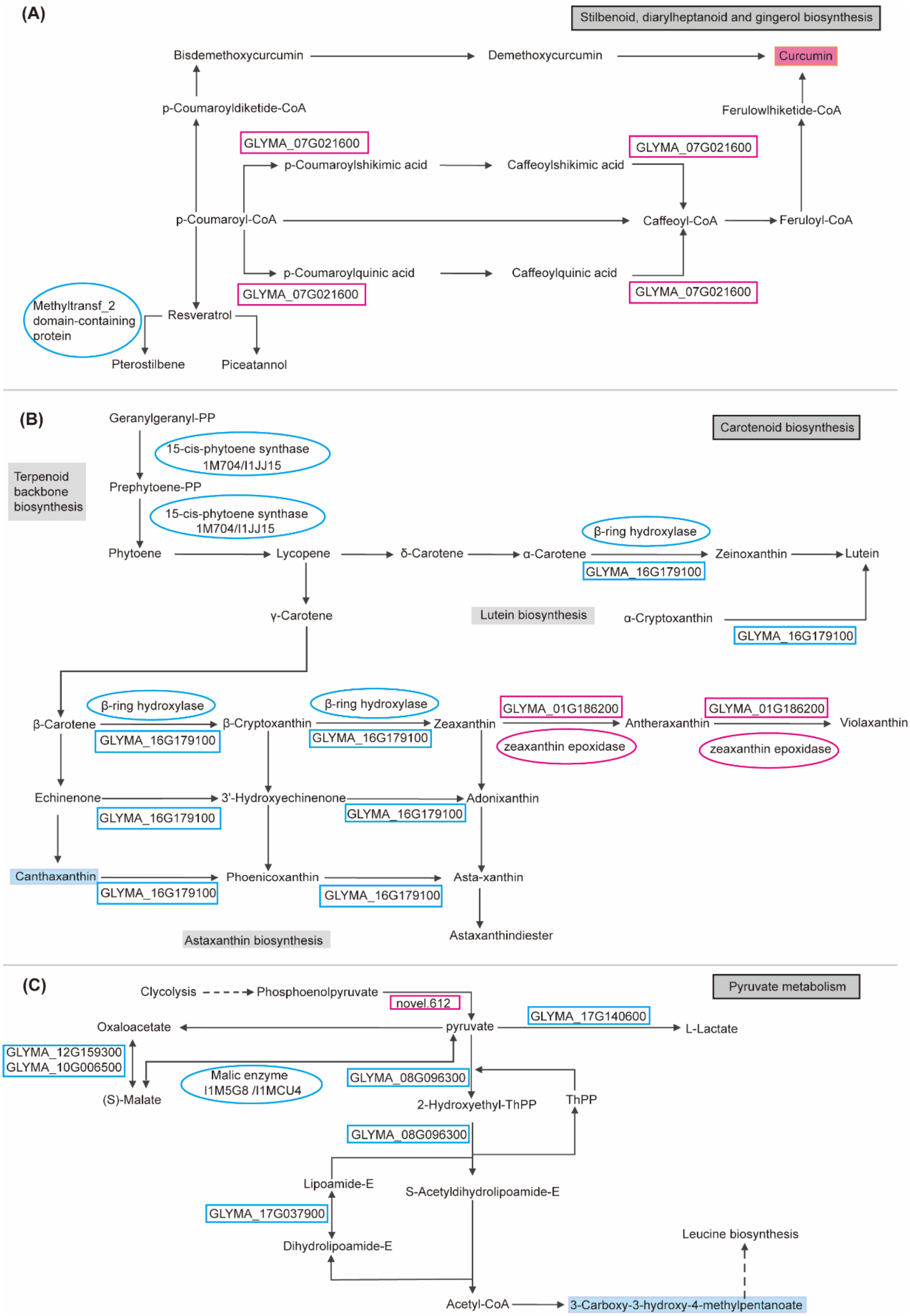

Figure S6. Integrated analysis of differentially expressed genes, proteins and metabolites in soybean under salt stress.

(A) Triomics identification of Stilbenoid, diarylheptanoid and gingerol biosynthesis

pathways.

(B) Triomics to identify changes in Carotenoid biosynthesis pathways.

(C) Triomics to identify changes in Pyruvate metabolism pathways.

Boxes indicate genes, round boxes indicate proteins, and underlined boxes are metabolites. Blue color indicates down-regulation and red color indicates up-regulation.

Figure S7

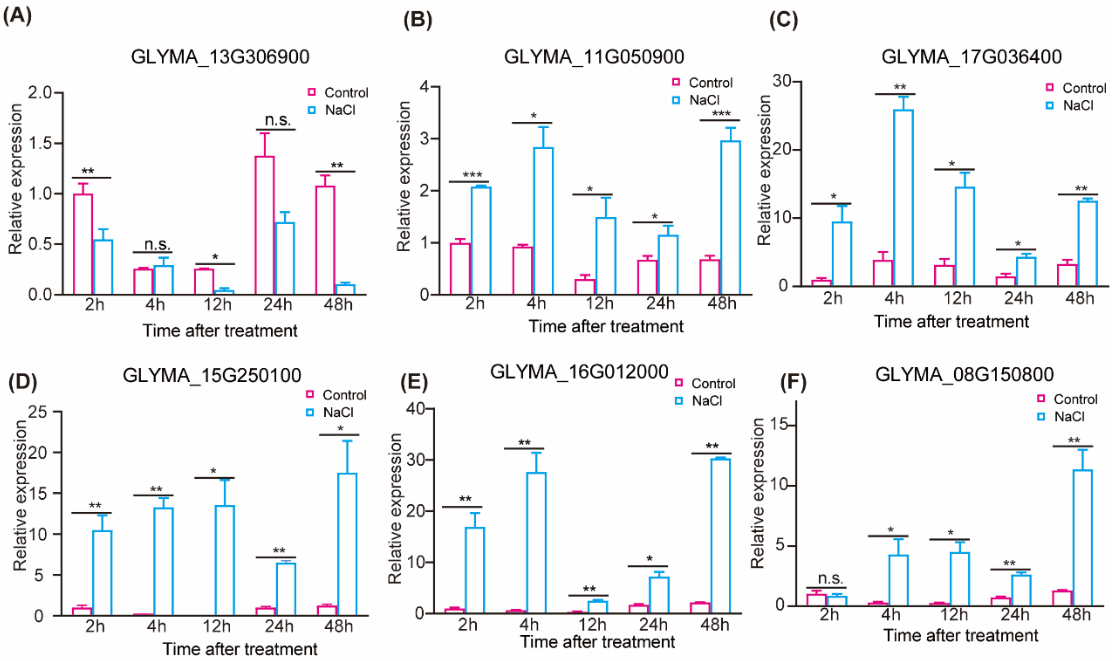

Figure S7. RT-qPCR validation of DEGs.

The relative expression levels of genes at different stages of 300 mM NaCl treatment. Each bar represents the average SD ±, n = 3. (t test, *: p ≤ .05; **: p ≤ .01; ***: p≤0.005; ****: p≤0.0001)

(A-F) RT-qPCR validation of DEGs (A, GLYMA_13G306900; B, GLYMA_11G050900; C, GLYMA_17G036400; D, GLYMA_15G250100; E, GLYMA_16G012000; F, GLYMA_08G150800). The relative expression levels of genes at different stages of 300 mM NaCl treatment. Each bar represents the average SD ±, n = 3. (t test, *: p ≤ 0.05; **: p ≤ 0.01; ***: p≤0.005; ****: p≤0.001)

Figure S8

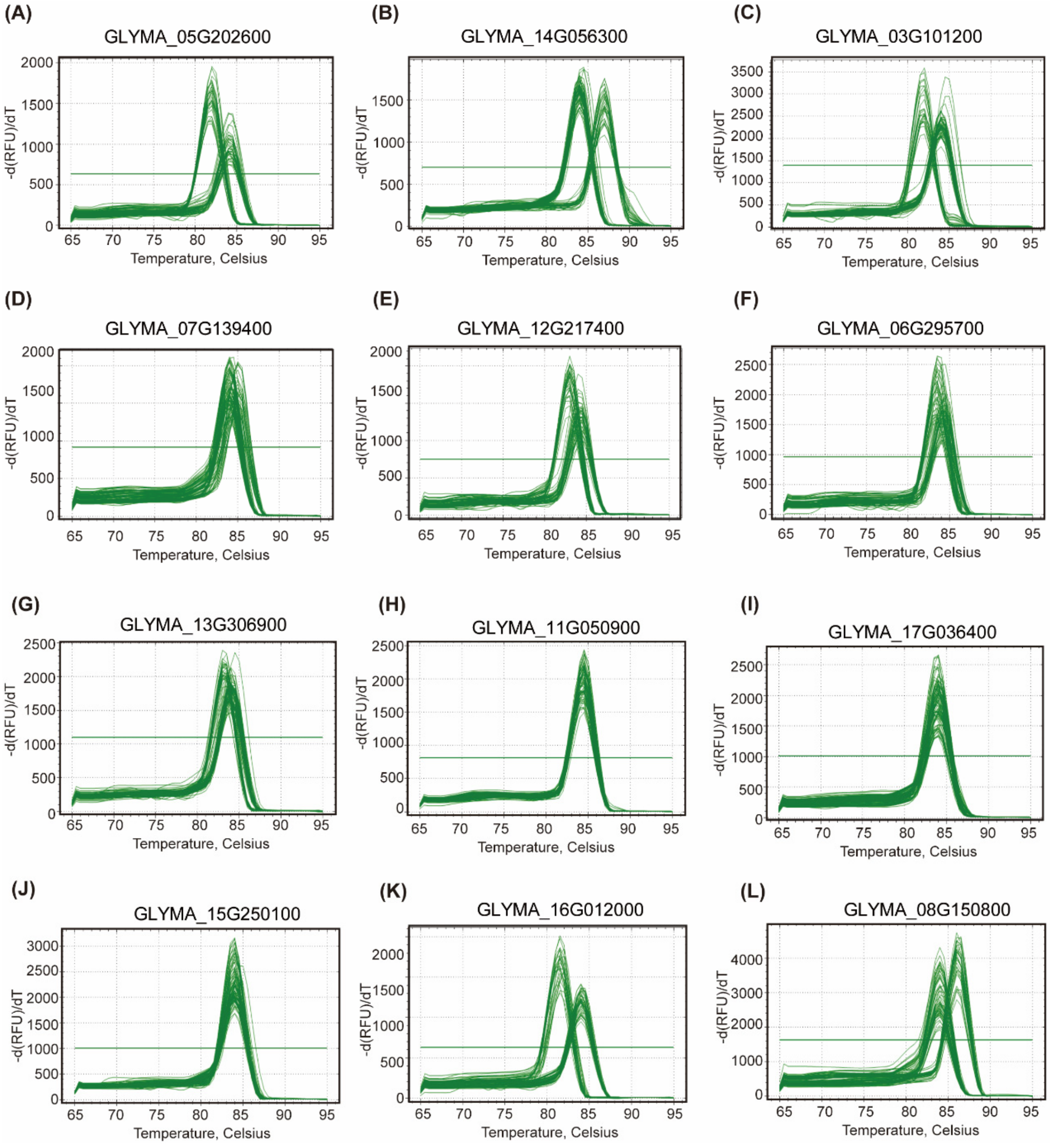

Figure S8. Real-time PCR melting curves.

(A-L) Melting curve of the gene of interest. The ordinate is the derivative (-dI/dT) of the change in temperature versus the fluorescence signal. The abscissa is the temperature.
