# Supplementary material for: Integrated Transcriptomic, Proteomic, and Metabolomic Analyses Revealed Molecular Mechanism for Salt Resistance in Soybean (Glycine max L.) Seedlings
Source: Int J Mol Sci. 2024 Dec 18;25(24):13559. doi: 10.3390/ijms252413559 (PMC11678865; doi:10.3390/ijms252413559)
Supplement: Supplementary file 1 [file ijms-25-13559-s001.zip › Supplementary materials-Table.pdf]

Table S1: Summary of transcriptome sample information

Sample Name	Raw reads	Raw bases	Clean reads	Clean bases	Error rate	Total map rate	Unique map rate	%GC
CK_1	47301902	7.1G	45923450	6.89G	0.03	96.50%	92.95%	44.07
CK_2	50090792	7.51G	47822934	7.17G	0.03	92.87%	89.19%	43.75
CK_3	48906912	7.34G	47115432	7.07G	0.03	96.63%	92.26%	44.08
S_2h_1	46643752	7G	44733318	6.71G	0.03	96.57%	92.80%	43.92
S_2h_2	49334780	7.4G	47312284	7.1G	0.03	96.46%	92.59%	44.43
S_2h_3	42895808	6.43G	40268802	6.04G	0.03	96.69%	92.83%	44.21
S_4h_1	48954584	7.34G	46896634	7.03G	0.03	96.38%	92.86%	44.18
S_4h_2	48636276	7.3G	46238396	6.94G	0.03	96.41%	92.42%	44.69
S_4h_3	47913174	7.19G	45228528	6.78G	0.03	96.34%	92.63%	44.53
S_12h_1	49989552	7.5G	47423844	7.11G	0.03	96.77%	92.72%	44.16
S_12h_2	48649500	7.3G	45357334	6.8G	0.03	65.15%	62.59%	42.95
S_12h_3	48208450	7.23G	45608910	6.84G	0.03	96.58%	92.72%	44.09
S_24h_1	49075726	7.36G	45524598	6.83G	0.03	96.87%	93.72%	43.35
S_24h_2	44143116	6.62G	41433908	6.22G	0.03	96.28%	92.77%	43.28
S_24h_3	49051488	7.36G	46394264	6.96G	0.03	97.10%	93.65%	44.02
S_48h_1	46838362	7.03G	43688740	6.55G	0.03	97.12%	93.61%	44.42
S_48h_2	48091366	7.21G	44911192	6.74G	0.03	97.07%	93.94%	44.28
S_48h_3	48449910	7.27G	46084492	6.91G	0.03	96.69%	93.37%	43.63
Average	47954192	7.19G	45442614	6.82G	0.03	94.69%	91.09%	44.00

Sample: the name of the sample
Raw reads: the number of reads in the raw data
Raw bases: the base number of the raw data (raw base=raw reads*150bp)
Clean reads: the number of reads filtered from the original data
Clean bases: the number of bases filtered from the original data (clean base=clean reads*150bp)
Error rate: the overall sequencing error rate of the data
Total map: The number of reads mapped to the genome and its percentage
Unique map: The number and percentage of reads aligned to a unique location in the reference genome (for subsequent quantitative data analysis of reads)
%GC: The percentage of G and C in clean reads for the four bases

TableS24. DEGs, DEPs, and DEMs involved in common pathways under salt stress

Pathway name	DEGs	P-value	DEPs	P-value	DEMs	P-value
Carbon fixation in photosynthetic organisms	GLYMA_03G246300	0.0001205	I1M5G8	0.1162305	L-Aspartic acid	0.20588235
	GLYMA_13G336200		I1MCU4			
	GLYMA_11G111100					
	GLYMA_19G046600					
	GLYMA_07G182300					
	GLYMA_13G046200					
	GLYMA_12G037400					
	GLYMA_12G159300					
	GLYMA_19G046800					
	GLYMA_06G015900					
	GLYMA_15G038100					
	GLYMA_19G106800					
	GLYMA_08G165400					
	GLYMA_10G006500					
Pyruvate metabolism	GLYMA_17G140600	0.7435894	I1M5G8	0.1601887	2-Isopropylmalic acid	0.20588235
	GLYMA_17G037900		I1MCU4			
	GLYMA_08G096300					
	GLYMA_12G159300					
	GLYMA_10G006500					
	novel.612					
Thiamine metabolism	GLYMA_20G142000	0.7981176	I1N019	0.0950567	thiamine phosphate	0.20588235
Alanine, aspartate and glutamate metabolism	GLYMA_02G228100	0.5972927	I1LLM2	0.2860115	L-Aspartic acid	0.00726315
	GLYMA_05G007600				Citric acid	
	GLYMA_01G129400				L-Argininosuccinate	
Carotenoid biosynthesis	GLYMA_16G179100	0.6027924	I1L6D2	0.0000117	Canthaxanthin	0.39316239
	GLYMA_01G186200		I1M704			

			I1JJ15<br>G0Z350			
Stilbenoid, diarylheptanoid and gingerol biosynthesis	GLYMA_07G021600	0.7237149	K7M862	0.1213385	Curcumin	0.21978022

Pathway name: DEGs, DEPs, and DEMs are enriched into the KEGG (Kyoto Encyclopedia of Genes and Genomes pathway).

DEGs: Differentially expressed genes.

DEPs: Differentially expressed proteins.

DEMs: Differentially expressed metabolites.

P-value: The probability of obtaining results at least as extreme as the observed results of a statistical hypothesis test, assuming that the null hypothesis is correct.

DEPs: I1M5G8 (Malic enzyme), I1MCU4 (Malic enzyme), I1N019 (ThiC-associated domain-containing protein), I1LLM2 (Asparagine synthetase [glutamine-hydrolyzing], I1L6D2 (Protein LUTEIN DEFICIENT 5, chloroplastic), I1M704 (15-cis-phytoene synthase), I1JJ15 (15-cis-phytoene synthase), G0Z350 (Zeaxanthin epoxidase, chloroplastic), and K7M862 (Methyltransf_2 domain-containing protein.

Table S25. Primers used in this study

Gene ID	Primer name	sequence（5' to 3'）
Tubulin	Tubulin-2F	TTGCCACCATCAAGACTAAG
	Tubulin-2R	CAACATACCAGTGAACAAAAGC
GLYMA_05G202600	Glyma.05g202600.5 F	ACATAAAGACGGGCGAAATA
	Glyma.05g202600.5 R	GAGGAGGAGTGACAGAGTAATAGG
GLYMA_17G224900	Glyma.17g224900.1 F	GGAACGAAAGAGGGACGAAGA
	Glyma.17g224900.1 R	TTAGGCTCAGGTGGAGGCAAT
GLYMA_11G050900	Glyma.11g050900.1 F	CAGTGGGTTCGTGTTGTCT
	Glyma.11g050900.1 R	ACCTGAATAATGCCCTTGA
GLYMA_17G036400	Glyma.17g036400.1 F	TGGCTCAAATCATGGTCAA
	Glyma.17g036400.1 R	GGCAGTAACAGCAGCAACA
GLYMA_14G056300	Glyma.14g056300.1 F	CCCTCTTCAACTCCACCGATTC
	Glyma.14g056300.1 R	GGTGCCGAGCAACCATCTCC
GLYMA_13G306900	Glyma.13g306900.1 F	ATAAAGAGCCTTGTTGAAGC
	Glyma.13g306900.1 R	GCAAAGAGTGTCTGTAGCG
GLYMA_11G170300	Glyma.11g170300.1 F	TCCACAAGGCACCAAACAA
	Glyma.11g170300.1 R	TCCAACCATAGCCAACTCC
GLYMA_15G250100	Glyma.15g250100.2 F	TCCAGAAGCCTTACCTCAAA
	Glyma.15g250100.2 R	CAGGAACCGATCACAACAAT
GLYMA_03G101200	Glyma.03g101200.1 F	CTCTTTGACATCTTTCCTCCTC
	Glyma.03g101200.1 R	AATGTACGGCCATAAACCC
GLYMA_14G063800	Glyma.14g063800.1 F	TGGAGATCGAGGAGGAGGG
	Glyma.14g063800.1 R	TTGGGAACAGTGACAGTGAGC
GLYMA_16G012000	Glyma.16g012000.1 F	TTTCCTTCTTCTCGTGCTCCT
	Glyma.16g012000.1 R	TCCATCTTGACGCTTCCTTTT
GLYMA_07G139400	Glyma.07g139400.1 F	CAGCAGCACAAGATGAGAAT
	Glyma.07g139400.1 R	TCAAGGCAGAGTGTAAGGTC
GLYMA_12G217400	Glyma.12g217400.1 F	CTGGGAAAGAACATTCAAGTA
	Glyma.12g217400.1 R	ATAGGCAGTGTCCATAGATAAA
GLYMA_08G150800	Glyma.08g150800.1 F	ACCGAGAAGCACTCCACAG
	Glyma.08g150800.1 R	CCAACCAAATCCAGCACAT
GLYMA_06G295700	Glyma.06g295700.1 F	TCTTTCGGAGATTGTTAGGG
	Glyma.06g295700.1 R	GTGATCCACGATTATGACTTTT

Table S2: DEGs after 2 h of salt stress

Table S3: DEGs after 4 h of salt stress

Table S4: DEGs after 12 h of salt stress

Table S5: DEGs after 24 h of salt stress

Table S6: DEGs after 48 h of salt stress

Table S2-S6:

gene_id: Gene number
Sample: the normalized readcount value of each sample
group: the average readcount of each group
log_2FoldChange: the ratio of gene expression levels between the treatment group and the control group, and then processed by the shrinkage model of the difference analysis software, and finally the logarithm is taken with 2 as the base
pvalue: The value of the significance test
padj: p-value corrected for multiple hypothesis testing
gene_name: The name of the gene
gene_chr: The name of the chromosome on which the gene is located
gene_start: The starting position of the gene on the chromosome
gene_end: The location where the gene terminates on the chromosome
gene_strand: Information on the positive and negative strands of the chromosome where the gene is located
gene_length: Gene length, sum of all non-overlapping regions of the gene from gene initiation to termination
gene_biotype: Gene type, such as coding protein genes, long non-coding genes, etc
gene_description: Gene function description, the first &&& symbol is preceded by reference genome annotation information, the first &&& symbol is followed by the swissprot database annotation, and the second &&& symbol is followed by the PFAM database annotation
gene_tf_family: Gene transcription factor family annotation

Table S7: GO function enrichment of DEGs at different times

Table S8: GO (Gene ontology) analysis of DEGs

Category: GO database classification, including biological process BP, cell component CC, molecular function MF
GOID: GO number
Description: the description of the function corresponding to the GO ID
GeneRatio: The ratio of the number of differential genes annotated to the GO number to the total number of differential genes

BgRatio: The ratio of the number of background genes annotated to the GO number to the total number of background genes
pvalue: The p-value of the significance test
padj: p-value corrected for multiple hypothesis testing
geneID: The differential gene ID annotated to the GO number
geneName: The name of the differential gene annotated to the GO number
Count: The number of differential genes annotated onto the GO number
Up: The number of differential genes that are upregulated in relation to this term
Up_Gene_id: Differential gene ID that is upregulated in relation to this term
Down: The number of differential genes that are down-regulated in relation to the term
Down_Gene_id: Differential gene IDs that are downregulated in relation to this term

Table S9: DEGs were enriched in KEGG Pathway

KEGG ID: KEGG pathway number
Description: the function description corresponding to the KEGG pathway number
GeneRatio: The ratio of the number of differential genes annotated to the total number of differential genes annotated to the KEGG pathway number
BgRatio: The ratio of the number of background genes annotated to the total number of background genes annotated to the KEGG pathway number
pvalue: The p-value of the significance test
padj: p-value corrected for multiple hypothesis testing
geneID: The differential gene ID annotated to the KEGG pathway number
geneName: The name of the differential gene annotated to the KEGG pathway number
keggID: keggID of the differential gene annotated to the KEGG pathway number
Count: The number of differential genes annotated to the KEGG pathway number
Up: The number of differential genes that are upregulated in relation to this term
Up_Gene_id: Differential gene ID that is upregulated in relation to this term
Down: The number of differential genes that are down-regulated in relation to the term
Down_Gene_id: Differential gene ID of down-regulated gene associated with this term

Table S10: DEPs after 24 h of 300 mM NaCl

Compared Samples: the sample pairs compared to the former than the latter
Num. of Total Quant.: the protein identified together in the two groups of samples (the non-jointly identified protein cannot be determined to be up-down-regulated) regulated type: the type of protein regulation
fold-change: the threshold of the difference multiple

Table S11: GO (Gene ontology) analysis of DEPs

GO_ID: GO identification number
GO_Term: Description of the function of GO identification number
GO_Class: GO class (MF, CC, BP)
Pvalue: Pvalue of enrichment analysis
AdjustedPv: corrected Pvalue
x: the number of differential proteins associated with the GO

y: the number of background (all) proteins associated with the GO
the number of differential proteins annotated by n:GO
the number of background (all) proteins annotated by N:GO
GOlevl: the number of levels of the term in the GO database
ProtID: protein ID;

Table S12: DEPs were enriched in KEGG Pathway

MapID: the ID of the enriched KEGG Pathway
MapTitle: the name of the enriched KEGG Pathway
Pvalue: Pvalue of enrichment analysis
AdjustedPv: corrected Pvalue
x: the number of differentially related proteins associated with the pathway
y: the number of background (all) proteins associated with the pathway
n: the number of differential proteins of kegg annotations
N: number of background (all) proteins for kegg annotations
ProtID: list of enriched proteins;

Table S13: Domain identification of DEPs

IPR ID: The ID of the domain to which the enrichment is enriched
IPR Title: Description of the domain
Pvalue: Pvalue of enrichment analysis
Adjusted: Corrected Pvalue
X: The number of differential proteins associated with this domain
Y: The number of background (all) proteins associated with this domain
n: Number of differential proteins annotated by the domain
N: The number of background (all) proteins annotated by the domain
EnrichDirect:cEnrichment pointed, Over or Under
ProteinIDs: List of enriched proteins
Description: A description of each protein

Table S14: Protein-protein interaction analysis.

prot1: Protein 1
prot2: Protein 2
score: Protein 1 and protein 2 interaction scores

Table S15: Positive ion DEMs after 24 h of salt stress

Table S16: Negative ion DEMs after 24 h of salt stress

Table S15-S16:

First column: ID, metabolite ID;
The second column: Name, metabolite description;
Column 3: Formula, molecular formula of metabolites;

Column 4: Molecular Weight, relative molecular weight of metabolites;
Fifth column: RT [min], retention time;
Sixth column: m/z, mass-to-charge ratio;
Columns 7-9: Identification of the three databases;
Column 10: Class_I, first-level classification of metabolites;
Column 11: Class_II, secondary classification of metabolites;
Column 12: Class_III, tertiary classification of metabolites;
Column 13: Class_IV., fourth level classification of metabolites;
Column 14: FC, multiple of the difference for comparison;
Column 15: log2FC, which compares the log2 value to the multiple of the difference;
Column 16: Pvalue, compare vs. significance p-value;
Column 17: ROC, receiver operating characteristic curve area AUC value;
Column 18: VIP, Variable Importance Projection, to reflect the degree to which the quantitative value of each sample contributes to the difference, one General settings VIP>1;
Column 19: Up.Down, up or down;
Column 20 - Column 1 from the bottom: quantitative values of different samples;

Table S17: Positive ion DEMs were enriched in KEGG Pathway

Table S18: Negative ion DEMs were enriched in KEGG Pathway

Table S17-S18

MapID: The ID of the enriched KEGG Pathway
MapTitle: the name of the enriched KEGG Pathway
Pvalue: Pvalue of enrichment analysis
x: The number of differential metabolites associated with this pathway
y: The number of background (all) metabolites associated with the pathway
n: Number of differential metabolites annotated by KEGG
N: Number of background (all) metabolites for KEGG annotation
Enrich Direct: Enrichment points to, and over represents enrichment, i.e., x/n>y/N
Meta IDs: list of enriched metabolites
kegg_cpd_id: IDs of metabolites in the KEGG database

Table S19: Correlation analysis of DEGs with positive ion DEMs

Table S20: Correlation analysis of DEGs with negative ion DEMs

Table S21: Combined analysis of DEGs and DEPs

Gene_id: Gene name
Tran_id: The transcription ID corresponding to the gene name
Prot_id: The protein ID of the gene name
GO_fun: GO features are enriched with significant entries

Kegg: KEGG function enriches the entries with more significant results
Protein(log2fc): The log_2 logarithm of the differential fold of the significantly differentially expressed protein corresponding to the gene name
Protein(pval): The pvalue of the protein that was significantly differentially expressed by the corresponding gene name
Tran(log2fc): The log_2 logarithm of significantly differentially expressed transcript folds corresponding to gene name
Tran(pval): Significantly differentially expressed transcript pvalue corresponding to gene name

Table S22: Correlation analysis of DEPs with positive DEMs

Table S23: Correlation analysis of DEPs with negative ion DEMs

Table S19,20,22,23

Corr: Correlation coefficient
