# Supplementary figures and images for: Integrated Transcriptomic, Proteomic, and Metabolomic Analyses Revealed Molecular Mechanism for Salt Resistance in Soybean (Glycine max L.) Seedlings
Source: Int J Mol Sci. 2024 Dec 18;25(24):13559. doi: 10.3390/ijms252413559 (PMC11678865; doi:10.3390/ijms252413559)

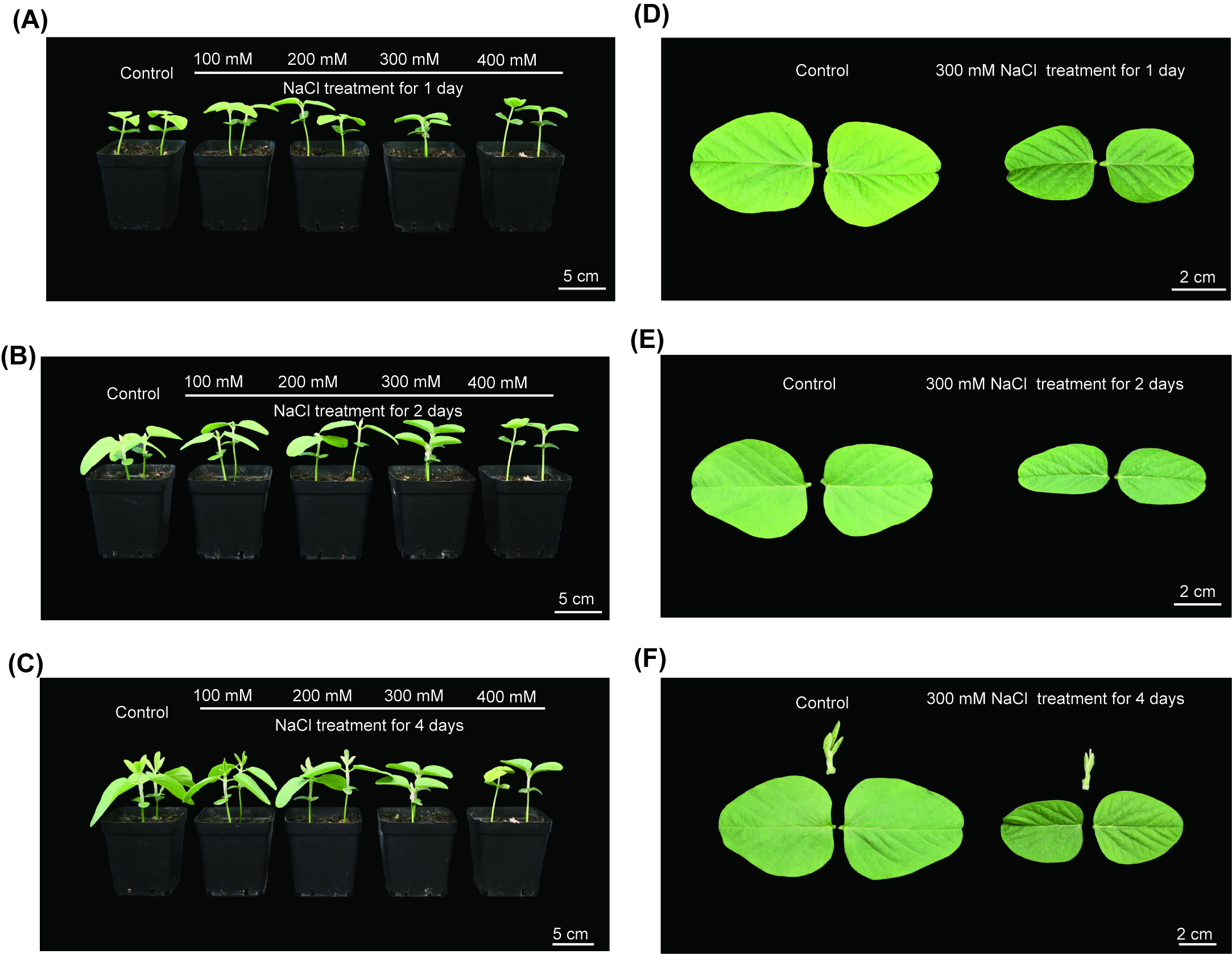

Supplement: Supplementary file 1 [file ijms-25-13559-s001.zip › Supplementary materials-Figure/Figure_S1.tif]

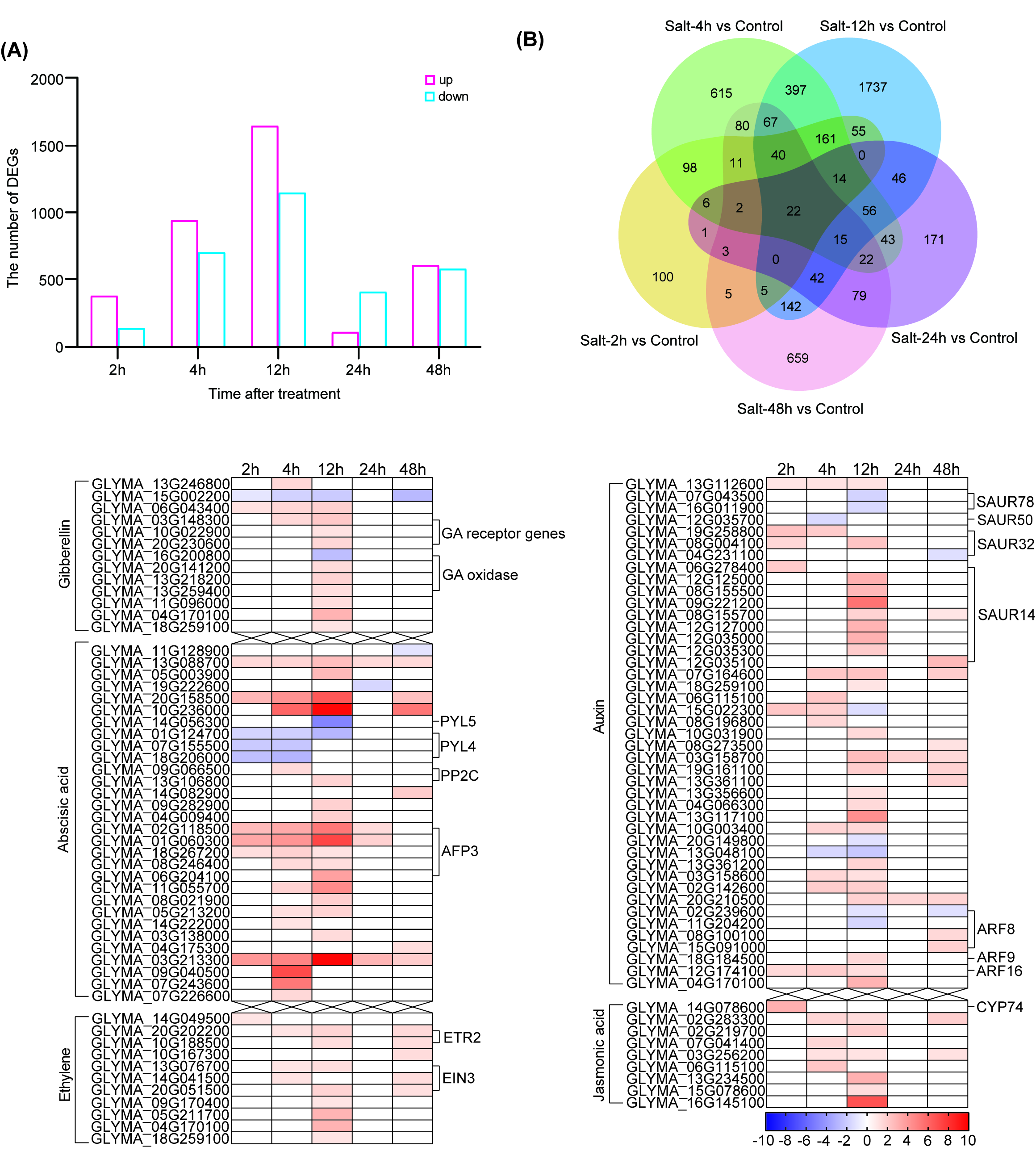

Supplement: Supplementary file 1 [file ijms-25-13559-s001.zip › Supplementary materials-Figure/Figure_S2.tif]

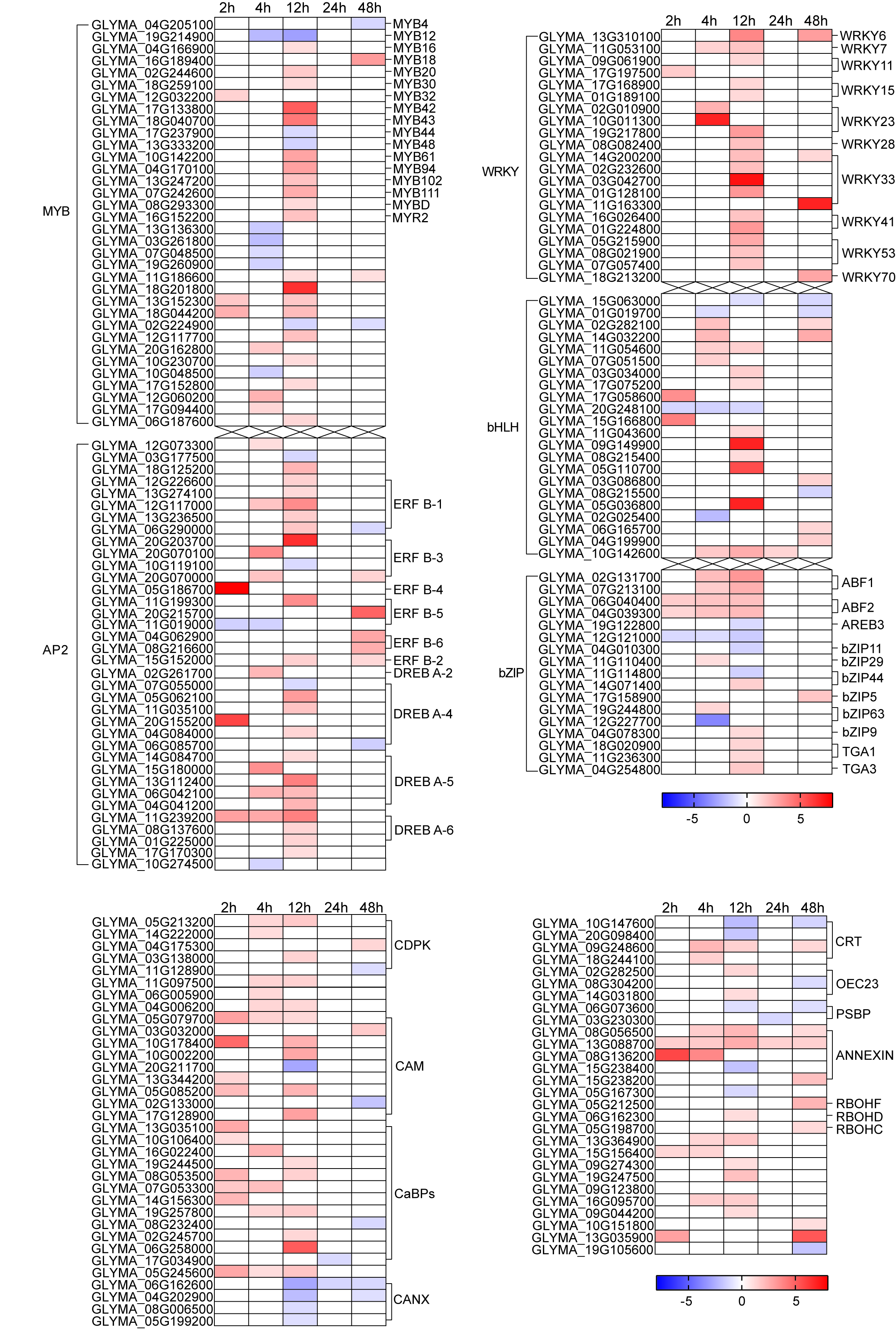

Supplement: Supplementary file 1 [file ijms-25-13559-s001.zip › Supplementary materials-Figure/Figure_S3.tif]

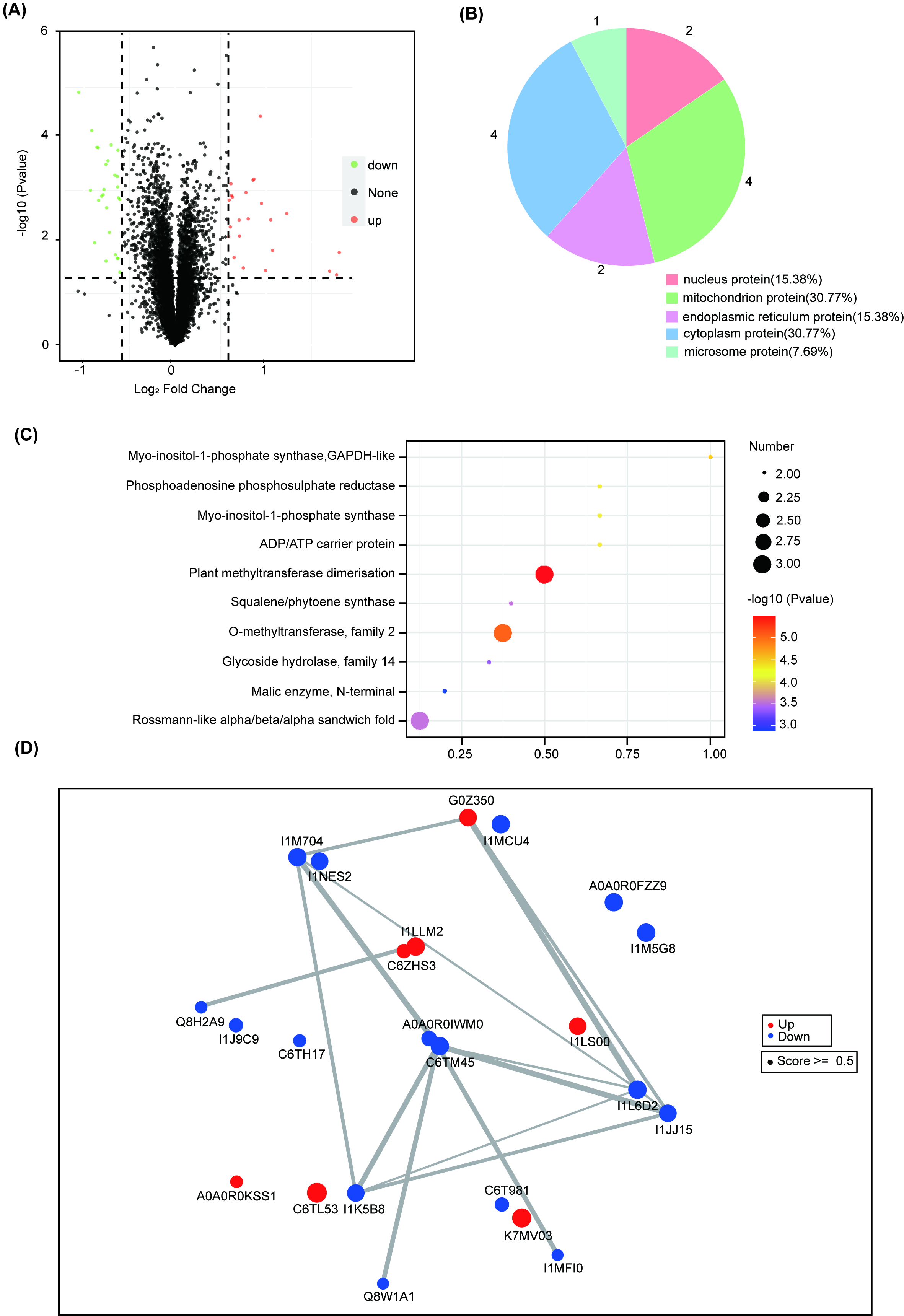

Supplement: Supplementary file 1 [file ijms-25-13559-s001.zip › Supplementary materials-Figure/Figure_S4.tif]

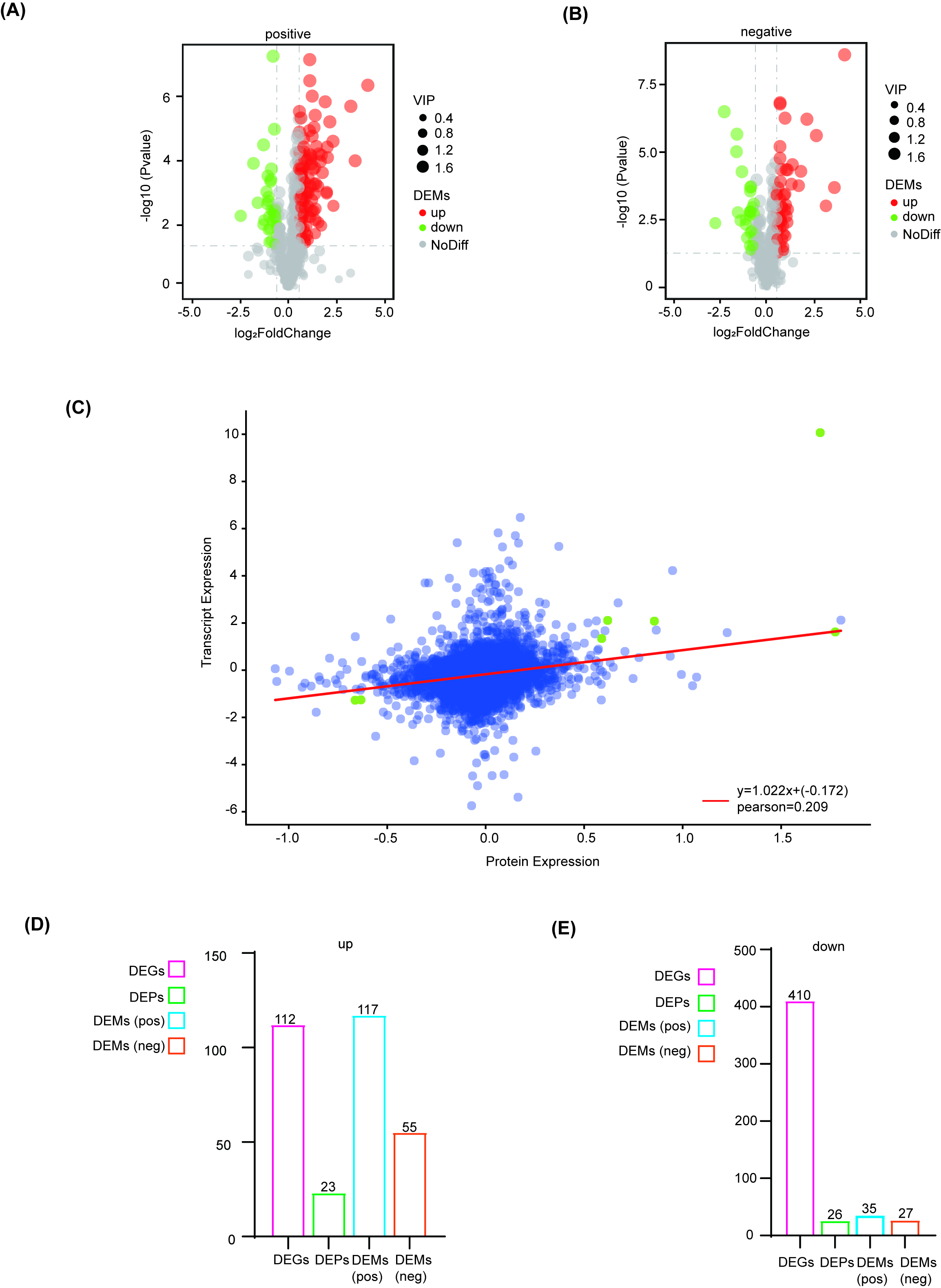

Supplement: Supplementary file 1 [file ijms-25-13559-s001.zip › Supplementary materials-Figure/Figure_S5.tif]

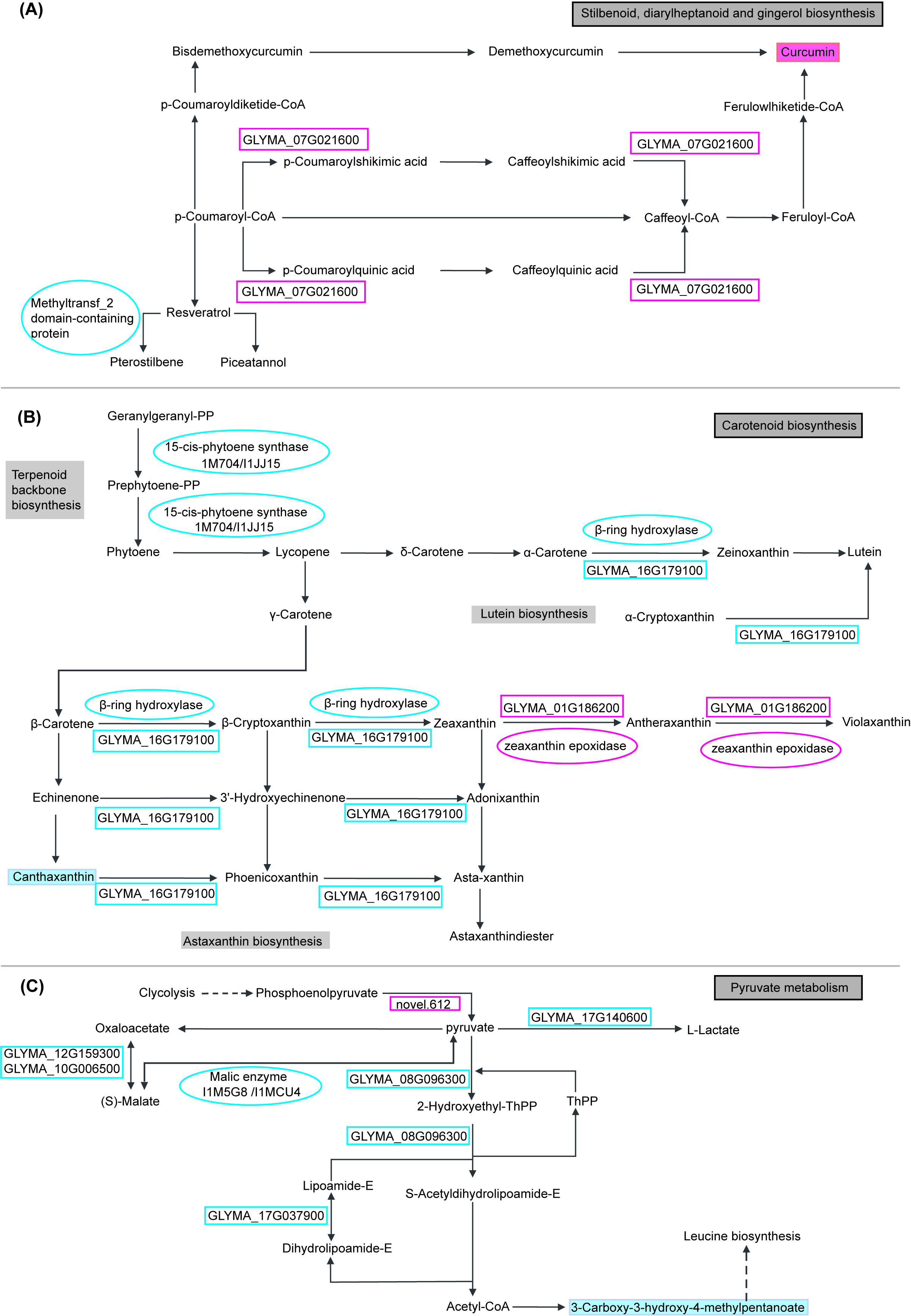

Supplement: Supplementary file 1 [file ijms-25-13559-s001.zip › Supplementary materials-Figure/Figure_S6.tif]

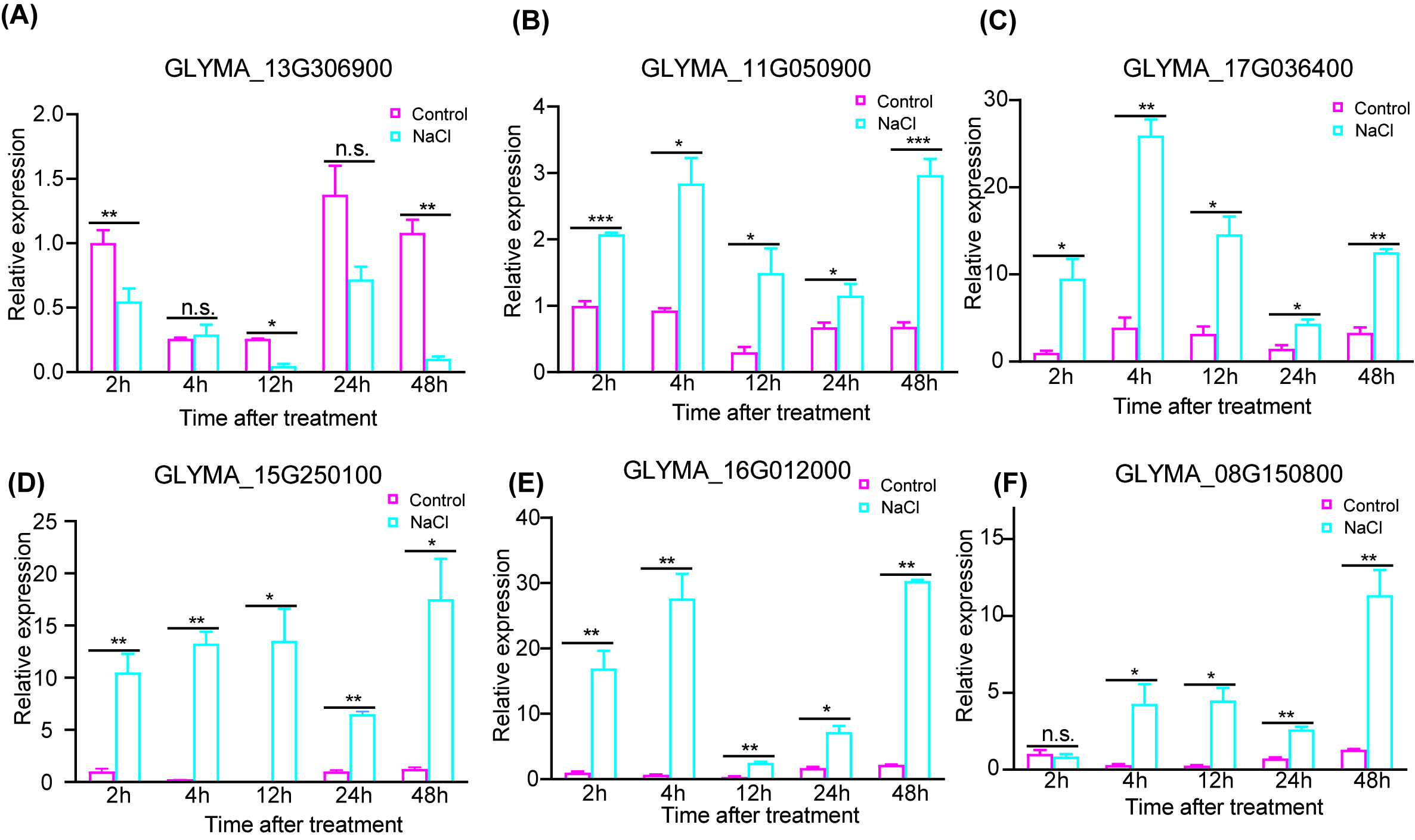

Supplement: Supplementary file 1 [file ijms-25-13559-s001.zip › Supplementary materials-Figure/Figure_S7.tif]

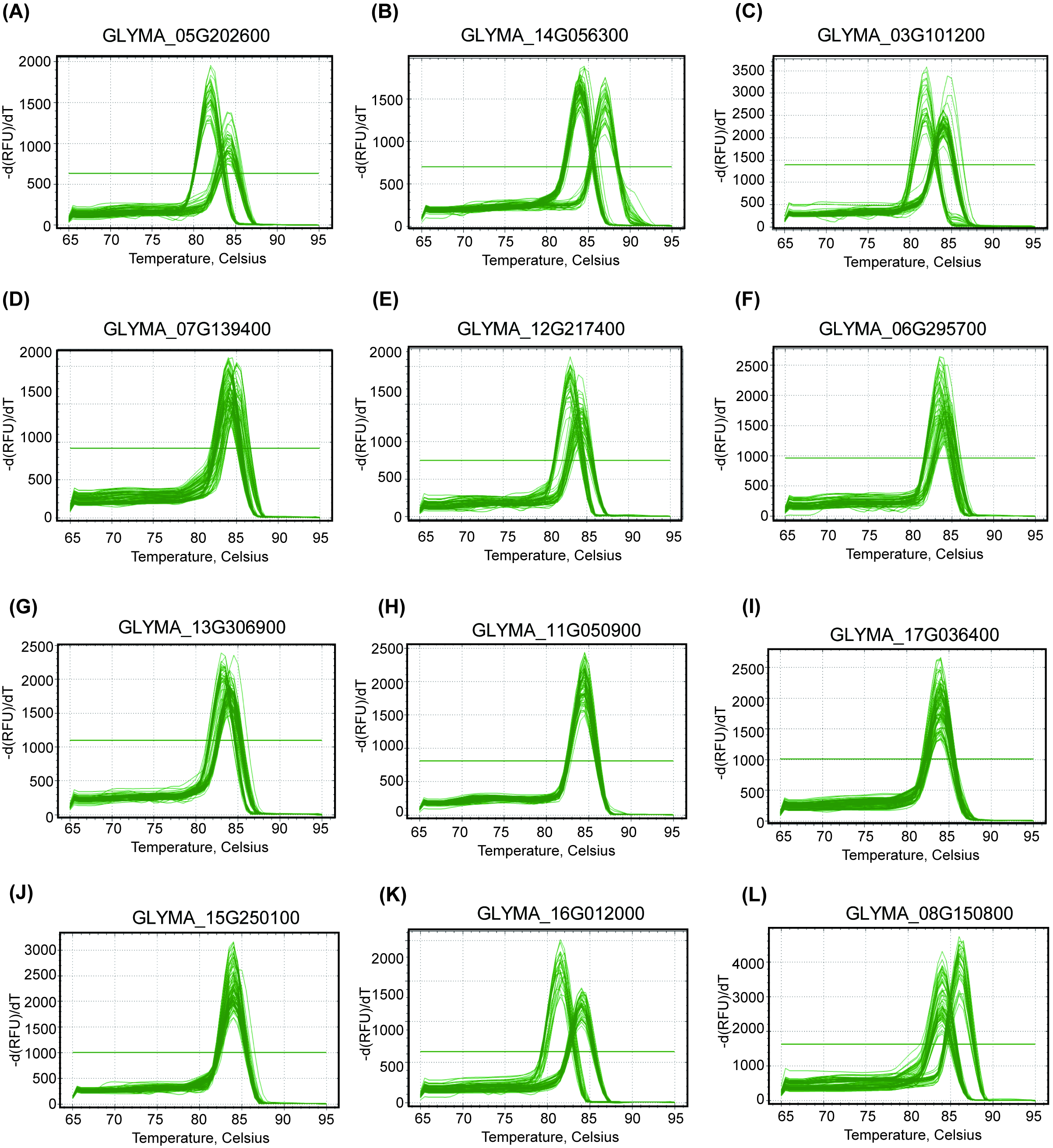

Supplement: Supplementary file 1 [file ijms-25-13559-s001.zip › Supplementary materials-Figure/Figure_S8.tif]
